# Acyl Chains of Phospholipase D Transphosphatidylation Products in Arabidopsis Cells: A Study Using Multiple Reaction Monitoring Mass Spectrometry

**DOI:** 10.1371/journal.pone.0041985

**Published:** 2012-07-25

**Authors:** Dominique Rainteau, Lydie Humbert, Elise Delage, Chantal Vergnolle, Catherine Cantrel, Marie-Anne Maubert, Sandrine Lanfranchi, Régis Maldiney, Sylvie Collin, Claude Wolf, Alain Zachowski, Eric Ruelland

**Affiliations:** 1 UPMC Univ Paris 06, ERL INSERM U 1057/UMR 7203, Paris, France; 2 UPMC Univ Paris 06, UR5, Physiologie Cellulaire et Moléculaire des Plantes, Paris, France; 3 CNRS, EAC7180, Paris, France; Max Planck Institute for Chemical Ecology, Germany

## Abstract

**Background:**

Phospholipases D (PLD) are major components of signalling pathways in plant responses to some stresses and hormones. The product of PLD activity is phosphatidic acid (PA). PAs with different acyl chains do not have the same protein targets, so to understand the signalling role of PLD it is essential to analyze the composition of its PA products in the presence and absence of an elicitor.

**Methodology/Principal findings:**

Potential PLD substrates and products were studied in *Arabidopsis thaliana* suspension cells treated with or without the hormone salicylic acid (SA). As PA can be produced by enzymes other than PLD, we analyzed phosphatidylbutanol (PBut), which is specifically produced by PLD in the presence of *n*-butanol. The acyl chain compositions of PBut and the major glycerophospholipids were determined by multiple reaction monitoring (MRM) mass spectrometry. PBut profiles of untreated cells or cells treated with SA show an over-representation of 160/18∶2- and 16∶0/18∶3-species compared to those of phosphatidylcholine and phosphatidylethanolamine either from bulk lipid extracts or from purified membrane fractions. When microsomal PLDs were used in *in vitro* assays, the resulting PBut profile matched exactly that of the substrate provided. Therefore there is a mismatch between the acyl chain compositions of putative substrates and the *in vivo* products of PLDs that is unlikely to reflect any selectivity of PLDs for the acyl chains of substrates.

**Conclusions:**

MRM mass spectrometry is a reliable technique to analyze PLD products. Our results suggest that PLD action in response to SA is not due to the production of a stress-specific molecular species, but that the level of PLD products *per se* is important. The over-representation of 160/18∶2- and 16∶0/18∶3-species in PLD products when compared to putative substrates might be related to a regulatory role of the heterogeneous distribution of glycerophospholipids in membrane sub-domains.

## Introduction

Phospholipase D (E.C. 3.1.4.4) catalyzes the hydrolysis of phospholipids to phosphatidic acid (PA) and alcohol. In eukaryotes, PLD activities play major roles in membrane trafficking and cell signalling [1], [2]. In plants, PLDs have been implicated in abiotic stress responses such as those caused by osmotic and salt stress [3], [4], heat [5] and cold [6]. PLDs are also activated in response to hormones, such as abscisic acid and salicylic acid (SA) [7], and to wounding and biotic elicitors [8]. Despite the physiological importance of these responses, relatively little is known about how PA acts. The signalling effects of PA have been suggested to derive from its ability to activate or deactivate proteins after recruiting them to membranes. In plants, PA has been shown to bind and activate NADPH-oxidases [9] thereby controlling abscisic acid-mediated stomatal closure. PA also binds and deactivates the phosphatase C2 ABI1 [10] and the MAPKK kinase CTR1 [11]. In animals, other PA targets have been identified, such as the TOR and Raf1 proteins [12].

PA is a generic term to describe a class of molecules that share the same glycerol-*sn-*3-phosphate structure, but that may be acylated at positions *sn*-1 and *sn*-2 of the glycerol moiety with a variety of fatty acids. Different PA molecules may therefore have different properties or signalling functions. For instance, Arabidopsis RbohD and RbohF NADPH oxidases bind *in vitro* to dioleoyl-PA (di18∶1-PA), dilinoleoyl-PA (di18∶2-PA), palmitoyl-/oleoyl-PA(16∶0/18∶1-PA), palmitoyl-/linoleoyl-PA (16∶0/18∶2-PA) and stearoyl-/linoleoyl-PA (18∶0/18∶2-PA) but not to the disaturated species dipalmitoyl-PA (di16∶0-PA) or distearoyl-PA (di18∶0-PA) [9]. By contrast di18∶2-PA does not bind sphingokinase1 but di16∶0-PA does [13]. As these differences in binding capacity are correlated with differences in the activation/inhibition of the protein targets [9], it is important to consider the fatty acid components of the PAs produced by PLDs when deciphering the signal transduction of specific stimuli.

Until recently, to discover the acyl chain composition of phospholipids it was necessary to first separate the different phospholipid classes by thin-layer chromatography, then separate in series the component methylated fatty acids by gas chromatography. However the development of mass spectrometry (MS)-based lipidomics methods now makes it possible to analyze the wide range of molecular species that constitute phospholipids more readily [14]–[19]. When tandem mass spectrometry is implemented with collision-induced dissociation (CID), phospholipid ions are accelerated through an inert gas atmosphere so fatty acids are cleaved into fragment-ions. Multiple reaction monitoring (MRM) can then be used to detect and quantify molecular species of glycerophospholipids by the acquisition of a pre-set list of ion masses *m/z*. The parent glycerophospholipid is detected in the first quadrupole (Q1) of the tandem MS, the second quadrupole (Q2) is the intermediary stage filled with inert gas where CID of the parent PA occurs, and the resultant fatty acid ion-fragments are detected in the third quadrupole (Q3). MRM has been used effectively to analyse glycerophospholipids from drosophila and yeast [20], [21].

PA can be produced in the cell by both PLD-dependent and PLD-independent pathways. PLDs have the unique capacity though to use primary alcohols as substrates in what is called the transphosphatidylation reaction, to produce a phosphatidylalcohol. A strategy to specifically monitor PLD-generated PA is to measure the amount of phosphatidylbutanol (PBut) formed in the presence of *n*-butanol [7], [18], [22].

The aim of this work was to investigate the nature of the PA signal elicited by SA by monitoring the product of the PLD transphosphatidylation reaction in Arabidopsis suspension cells. The compositional profile of the PBut product was analyzed by mass spectrometry in MRM mode and compared to the profiles of the major glycerophospholipid classes. We found that the acyl chain profile of PBut in cells challenged with or without SA does not match those of the main glycerophospholipid classes, suggesting that when PLD produces the PA signal *in vivo* there is some enrichment in molecules composed of specific acyl chains. Possible explanations of this specificity are discussed in terms of preferential PLD substrates and cell membrane heterogeneity *in vivo*.

## Results

### Mass Scanning of the Major Glycerophospholipids in Arabidopsis Cells

**Figure 1:**
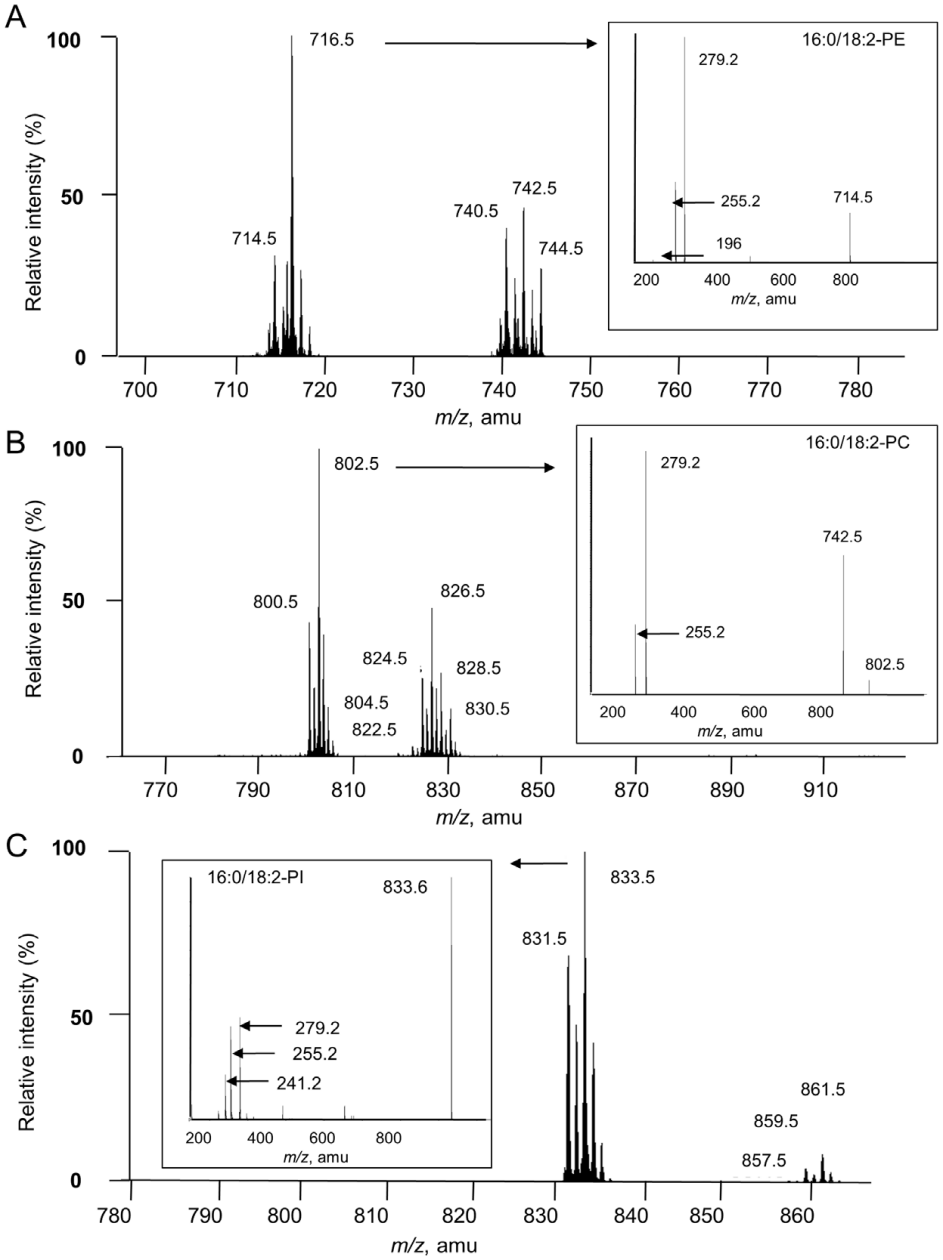
Mass scan of the main glycerophospholipids in suspension cells. Mass spectra of parent ions of phosphatidylethanolamine (A), phosphatidylcholine (B) and phosphatidylinositol (C) were recorded by the neutral loss of fragment 141 amu (from positive ions), the neutral loss of fragment 60 amu (from negative ions) and the precursors of fragment *m/z* 241 (from negative ions), respectively. Mass spectra correspond to the [M+H]^+^ ions for PE, [M+HCOO]^−^ for PC and [M−H]^−^ ions for PI. The glycerophospholipid fatty acid composition was determined in the negative mode by CID as shown (insert) for 16∶0/18∶2-molecular species.

To quantify molecular species of different glycerophospholipids by MRM, we selected the ion corresponding to the full phospholipid in the first quadrupole and one of its constituent fatty acids in the third quadrupole of the spectrometer. It was first necessary to establish a list of expected transitions (or couple of masses). The mass scan spectra of phosphatidylinositol (PI), phosphatidylcholine (PC) and phosphatidylethanolamine (PE), the main classes of glycerophospholipid in plants, were obtained by precursor ion scanning at *m/z* 241 (negative mode), by neutral loss scan of 60 amu (negative mode) and neutral loss scan of 141 amu (positive mode), respectively [17] (Figure 1). The masses for PE, PC and PI correspond to the [M+H]^+^ ions, the [M+HCOO]^−^ ions and the [M-H]^-^ ions, respectively. The glycerophospholipid fatty acid composition was determined in the negative mode by collision induced dissociation (CID) as shown (insert) for 16∶0/18∶2-molecular species. The insert spectra show the prominence of *m/z* 279 (18∶2 fatty acid) relatively to *m/z* 255 peak (corresponding to 16∶0). This is due to the favored cleavage from *sn*-2 position of the glycerol moiety that is explained by the shorter range from the anionic charge of phosphate [23]. PC and PE were also detected by the scanning of precursors of fragment ions *m/z* 184 (in the positive mode) and fragment ions *m/z* 196 (in the negative mode), respectively. Mass spectra consistent with the alternative ionization mode confirmed the correct assignment of PC and PE structures (data not shown).

For a given glycerophospholipid class as defined by the polar head group, a single mass may correspond to various combinations of fatty acids, i.e. multiple molecular species (Table 1). For instance, *m/z* 826.5 in the PC class corresponds to a total of 36 carbon atoms and 4 unsaturated bonds in the two fatty acids (36∶4-PC), which can be assigned to either 18∶2/18∶2-PC or 18∶1/18∶3-PC. These two molecular species can therefore be distinguished by analyzing the product ions. The transitions that take into account all possible fatty acyl moieties cleaved from the parent ions by CID are listed in Table 1. Transitions corresponding to phosphatidylglycerol (PG) specific molecular species (16∶1/18∶1-, 16∶1/18∶2- and 16∶1/18∶3-PG) [23], [24], [25] are also included in the list since PG is a major glycerophospholipids in chloroplast membranes and may be a PLD substrate. However no neutral loss or precursor ion scanning is specific for PG.

**Table 1:** List of MRM transitions.

			*m/z* (amu)									
			PC		PE		PI		PG		PBut	
transitions			Q1	Q3	Q1	Q3	Q1	Q3	Q1	Q3	Q1	Q3
16∶0/18∶1	a		**804.5 →** 281.2		**716.5 →** 281.2		**835.5 →** 281.2		747.5 → 281.2		729.6 → 281.2	
	b		**804.5 →** 255.2		**716.5 →** 255.2		**835.5 →** 255.2		747.5 → 255.2		729.6 → 255.2	
16∶0/18∶2	a		**802.5 →** 279.2		**714.5 →** 279.2		**833.5 →** 279.2		745.5 → 279.2		727.6 → 279.2	
	b		**802.5 →** 255.2		**714.5 →** 255.2		**833.5 →** 255.2		745.5 → 255.2		727.6 → 255.2	
16∶0/18∶3	a		**800.5** → 277.2		**712.5** → 277.2		**831.5** → 277.2		743.5 → 277.2		725.6 → 277.2	
	b		**800.5** → 255.2		**712.5** → 255.2		**831.5 →** 255.2		743.5 → 255.2		725.6 → 255.2	
16∶1/18∶1	a		**802.5 →** 253.2		**714.5 →** 253.2		**833.5 →** 253.2		745.5 → 253.2		727.6 → 253.2	
	b		**802.5 →** 281.2		**714.5 →** 281.2		**833.5 →** 281.2		745.5 → 281.2		727.6 → 281.2	
16∶1/18∶2	a		**800.5** → 279.2		**712.5** → 279.2		**831.5** → 279.2		743.5 → 279.2		725.6 →279.2	
	b		**800.5** → 253.2		**712.5** → 253.2		**831.5** → 253.2		743.5 → 253.2		725.6 → 253.2	
16∶1/18∶3	a		798.5 → 277.2		710.5 → 277.2		829.5 → 277.2		741.5 → 277.2		723.6 →277.2	
	b		798.5 → 253.2		710.5 → 253.2		829.5 → 253.2		741.5 → 253.2		723.6 → 253.2	
18∶0/18∶1	a		832.6 → 281.2		744.6 **→** 281.2		863.6 → 281.2		775.6 → 281.2		757.6 → 281.2	
	b		832.6 → 283.2		744.6 **→** 283.2		863.6 → 283.2		775.6 → 283.2		757.6 →283.2	
18∶0/18∶2	a		**830.6 →** 279.2		**742.6 →** 279.2		**861.6 →** 279.2		773.6 → 279.2		755.6 → 279.2	
	b		**830.6 →** 283.2		**742.6 →** 283.2		**861.6 →** 283.2		773.6 → 283.2		755.6 → 283.2	
18∶0/18∶3	a		**828.5 →** 277.2		**740.5 →** 277.2		**859.5 →** 277.2		771.5 → 277.2		753.6 → 277.2	
	b		**828.5 →** 283.2		**740.5 →** 283.2		**859.5 →** 283.2		771.5 → 283.2		753.6 → 283.2	
18∶1/18∶1			**830.6 →** 281.2		**742.6 →** 281.2		**861.6 →** 281.2		773.6 → 281.2		755.6 → 281.2	
18∶1/18∶2	a		**828.5 →** 279.2		**740.5 →** 279.2		**859.5 →** 279.2		771.5 → 279.2		753.6 → 279.2	
	b		**828.5 →** 281.2		**740.5 →** 281.2		**859.5 →** 281.2		771.5 → 281.2		753.6 → 281.2	
18∶1/18∶3	a		**826.5 →** 281.2		**738.5 →** 281.2		**857.5 →** 281.2		769.5 → 281.2		751.6→ 281.2	
	b		**826.5 →** 277.2		**738.5 →** 277.2		**857.5 →** 277.2		769.5 → 277.2		751.6 → 277.2	
18∶2/18∶2			**826.5 →** 279.2		**738.5 →** 279.2		**857.5 →** 279.2		769.5 → 279.2		751.6 → 279.2	
18∶2/18∶3	a		**824.5 →** 277.2		**736.5 →** 277.2		**855.5 →** 277.2		767.5 → 277.2		749.6 → 277.2	
	b		**824.5 →** 279.2		**736.5 →** 279.2		**855.5 →** 279.2		767.5 → 279.2		749.6 → 279.2	
18∶3/18∶3			**822.5 →** 277.2		734.5 → 277.2		853.5 **→** 277.2		765.5 → 277.2		747.6 → 277.2	

The list was established from PC, PI and PE enhanced resolution mass spectra. From the spectra the molecular species can be deduced as “carbon number:double bond”. The detailed fatty acid composition of the molecular species consistent with carbon number:double-bond formula can be assayed by transitions parent → product ions after CID of the ester bonds. In bold: *m/z* detected for PE, PC and PI by enhanced-resolution mass scanning. Underlined: transitions detected by product ion analysis. For PE and PI the parent ions *m/z* correspond to the [M−H]^−^ and for PC it corresponds to [M+HCOO]^−^. In agreement with formula a difference of 2 amu is noted between *m/z* of PE MRM (negative ionization M−H) and neutral loss of *m/z* 141 (positive ionization M+H).

### Multiple Reaction Monitoring of the Major Glycerophospholipids in Arabidopsis Cells

**Figure 2:**
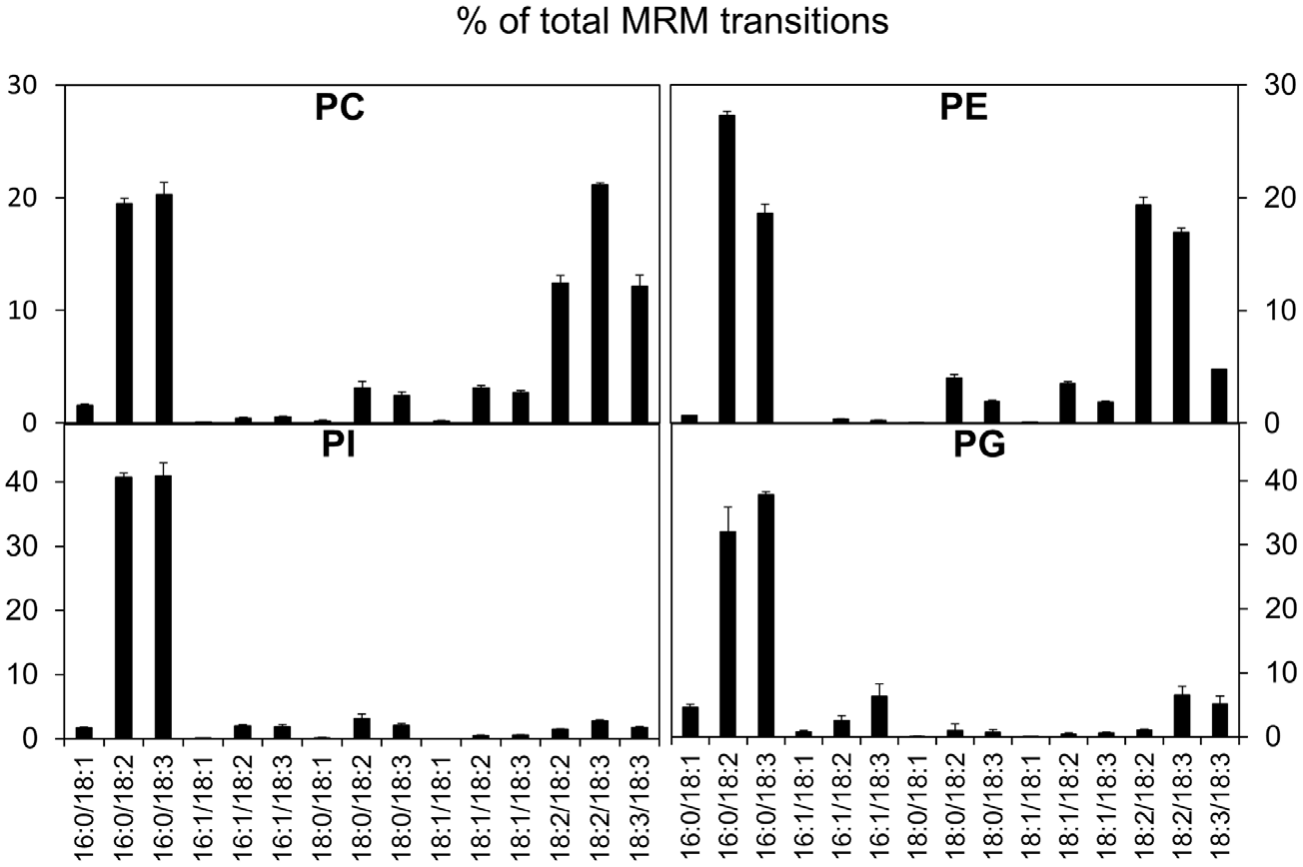
Analysis of the main glycerophospholipids in Arabidopsis cells by MRM mass spectrometry. Lipids were analyzed, searching for the transitions listed in Table 1.

The lipids from an Arabidopsis cell homogenate were analyzed by MRM using the transitions shown in Table 1 (Figure 2). Representative spectrum of the MRM transitions are shown in figure S1, using PI as an example. Notably *trans*-3-hexadecenoic (16∶1) fatty acid, which is characteristic of PG from *Arabidopsis thaliana*, is found as 16∶1/18∶3-, 16∶1/18∶2- and 16∶1/18∶1-molecular species, which are virtually absent in the other glycerophospholipid classes. For each glycerophospholipid class the two major molecular species are 16∶0/18∶2 and 16∶0/18∶3 in different proportions. Another striking difference is in the relative amounts of dipolyunsaturated fatty acyl (diPUFA) species. They are absent in PI and are a very minor component of PG, but in PE and PC they account for 40% or more of all molecular species. In PE the most abundant diPUFA species is di18∶2 followed by 18∶2/18∶3 then di18∶3, while in PC 18∶2/18∶3 is the most abundant diPUFA species.

### Validation of MRM as a Method to Analyze Phosphatidylbutanol Composition

To verify that it was possible to analyze the composition of PBut acyl chains by MRM, PBut was generated by the action of purified cabbage PLD. Substrates PE and PC purified from either Arabidopsis (Figure S2) or from soybean (data not shown) were used in the two-phase *in vitro* PLD reaction in the presence of 1.2% (v/v) *n*-butanol. In each case, as expected, the molecular species profile of the PBut produced matched that of the substrate provided. These results validated our approach showing that it is possible to detect PBut and distinguish its acyl composition by MRM.

### In vivo PLD Activity Triggered by Salicylic Acid Leads to a Product with a Specific Fatty Acid Composition

**Figure 3:**
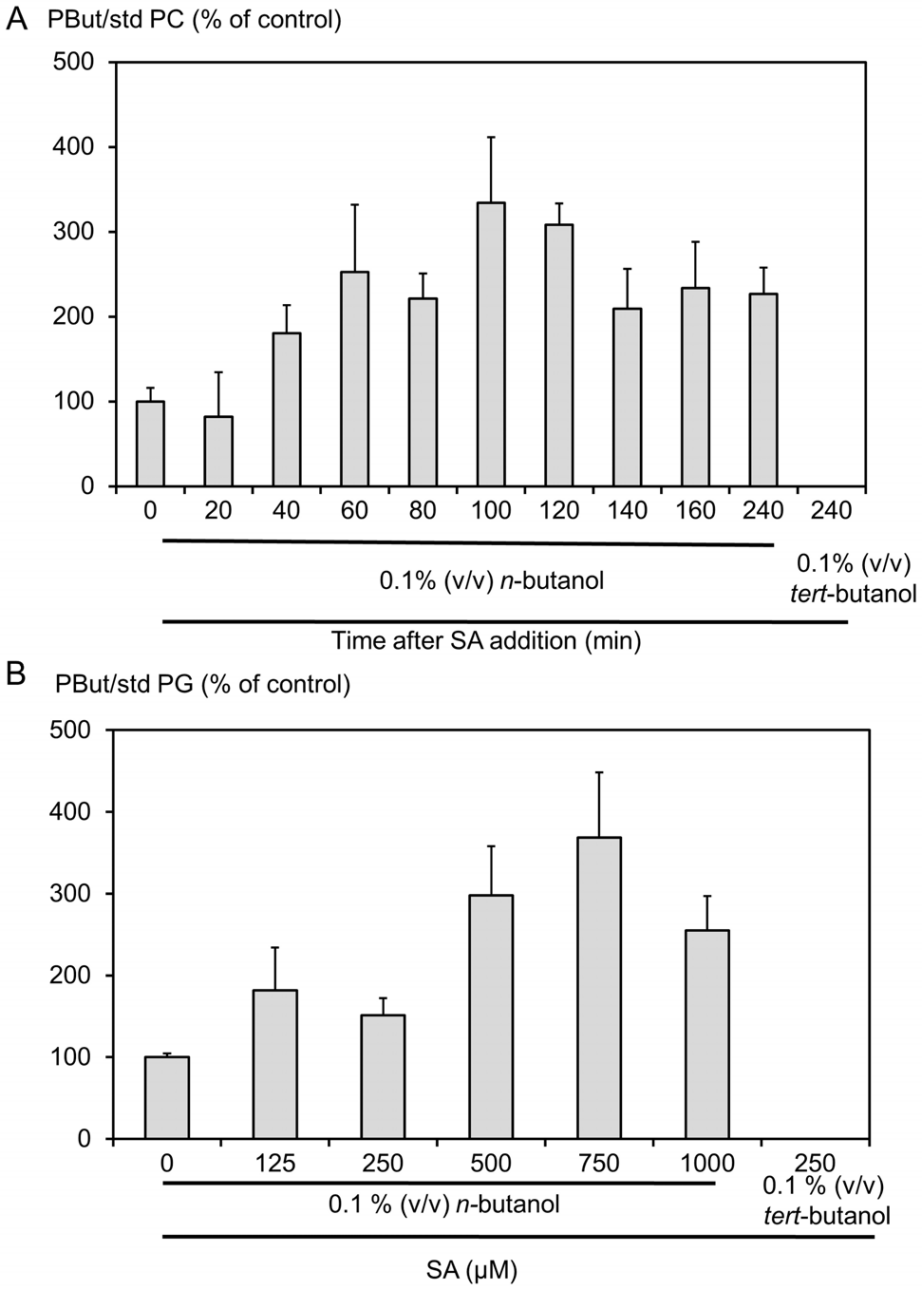
PLD is activated upon SA stimulation. Cell medium was supplemented with 0.1% (v/v) *n*-butanol and forty-five minutes later SA was added. Lipids were extracted at different times after cells were treated with 750 µM SA (A) or at 80 min after the addition of SA at different concentrations (B). Lipids were analyzed by mass spectrometry in the MRM mode, searching for the transitions listed in Table 1. The sum of the areas of the peaks corresponding to transitions for PBut was normalized to the area of the peak corresponding to the transition corresponding to 14∶0/14∶0-PC or to 14∶0/14∶0-PG. The levels of PBut are expressed as percentage of that in the control.

Arabidopsis cells in suspension culture were stimulated with SA in the presence of *n*-butanol. The production of PBut, as measured by the sum of all corresponding MRM transitions, was normalized relative to an internal standard, either dimyristoyl-PG or -PC. When cells were challenged with 750 µM SA in the presence of 0.1% (v/v) *n-*butanol, an increase in PBut was detected after 40 min and the amount of PBut produced reached a plateau 60 min after SA addition that was 2.5 fold higher than in unchallenged cells (Figure 3A). The effect of SA concentration on PLD activation was assayed 80 min after the addition of SA to cells. The response was dose-dependent with a maximum at 750 µM SA and was inhibited at a higher SA concentration (Figure 3B). *tert*-butanol, which is not a substrate for PLD in the transphosphatidylation reaction, was used in the control and as expected no signal corresponding to PBut was detected. The time-course and dose-response kinetics of SA-dependent activation of PLD observed by mass scanning are very similar to those obtained by ^33^P-orthophosphate labelling of glycerophospholipids [7].

**Figure 4:**
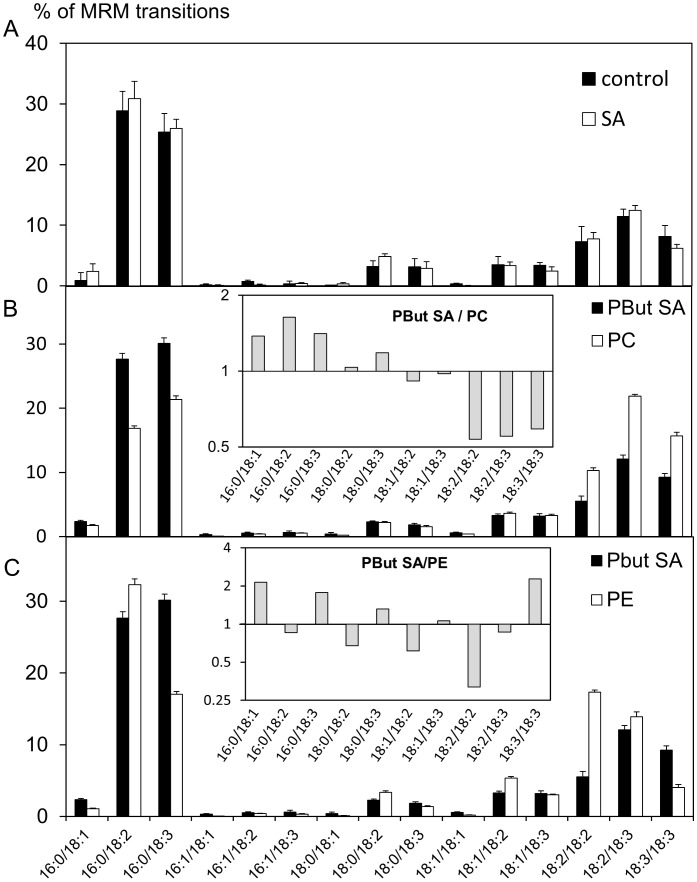
PBut profiles analyzed before and after SA addition. Cell medium was supplemented with 0.1% (v/v) *n*-butanol. After 45 minutes, 750 µM SA was added, and lipids were extracted 100 min later. (A) Black bars, PBut in untreated cells; white bars, PBut in SA treated cells; striped bars, PC; grey bars, PE. (B) PBut in the presence of SA compared to PC. Black bars, PBut in presence of SA; white bars, PC. Insert: for molecular species representing more than 1% of the species of PBut and PC, we calculated the ratio of the value obtained in PBut to the value obtained in PC. Results are represented on a *log2* scale. (C) PBut in the presence of SA compared to PE. Black bars, PBut in the presence of SA; white bars, PE. Insert: for molecular species representing more than 1% of the species of PBut and PE, we calculated the ratio of the value obtained in PBut to the value obtained in PE. Results are represented on a *log2* scale.

The molecular species of PBut were analyzed before and after cells had been treated with 750 µM SA for 100 min. The acyl chain profiles of PBut were compared to those of the major glycerophospholipid classes prepared from the same bulk lipid extract (Figure 4). PBut profiles were also established after 80 min of treatment with different SA concentrations (125 to 1000 µM, Figure S3A), or after different times (60, 100, 120 and 240 min) after treatment with 750 µM SA (Figure S3B). Firstly, all the PBut fatty acid profiles are similar under the different conditions tested. Secondly, they consistently show enrichment in 16∶0/18∶3-species compared to PC or PE profiles. This species represents 25–30% of all species in PBut but only 20% in PC (Figure 4B) and 18% in PE (Figure 4C). The relative proportion of 16∶0/18∶2-species in the PBut profiles (28–30%) is also higher than in PC (18%). For molecular species representing more than 1% of PBut, PC or PE species, we calculated the ratio of the value obtained for PBut to that obtained for PC (insert in figure 4B) or for PE (insert in figure 4C). PBut is overall more similar to PC, but with a relative enrichment in the saturated 16∶0/18∶1, 16∶0/18∶2 and 16∶0/18∶3 species correlating with a depletion in diPUFA species. The description of the relationships between PBut to PE ratios is not straightforward. In all case, this result means that it is not possible to directly deduce which phospholipids are *in vivo* PLD substrates.

### In vitro Arabidopsis PLD Product Profiles Match those of Glycerophospholipid Substrates

The striking difference between PLD substrate and product profiles could result from PLD discriminating between molecular species of a substrate. To investigate this, the molecular species profiles of PC and PE were compared to PBut produced *in vitro* from the respective substrates by Arabidopsis PLDs.

**Figure 5:**
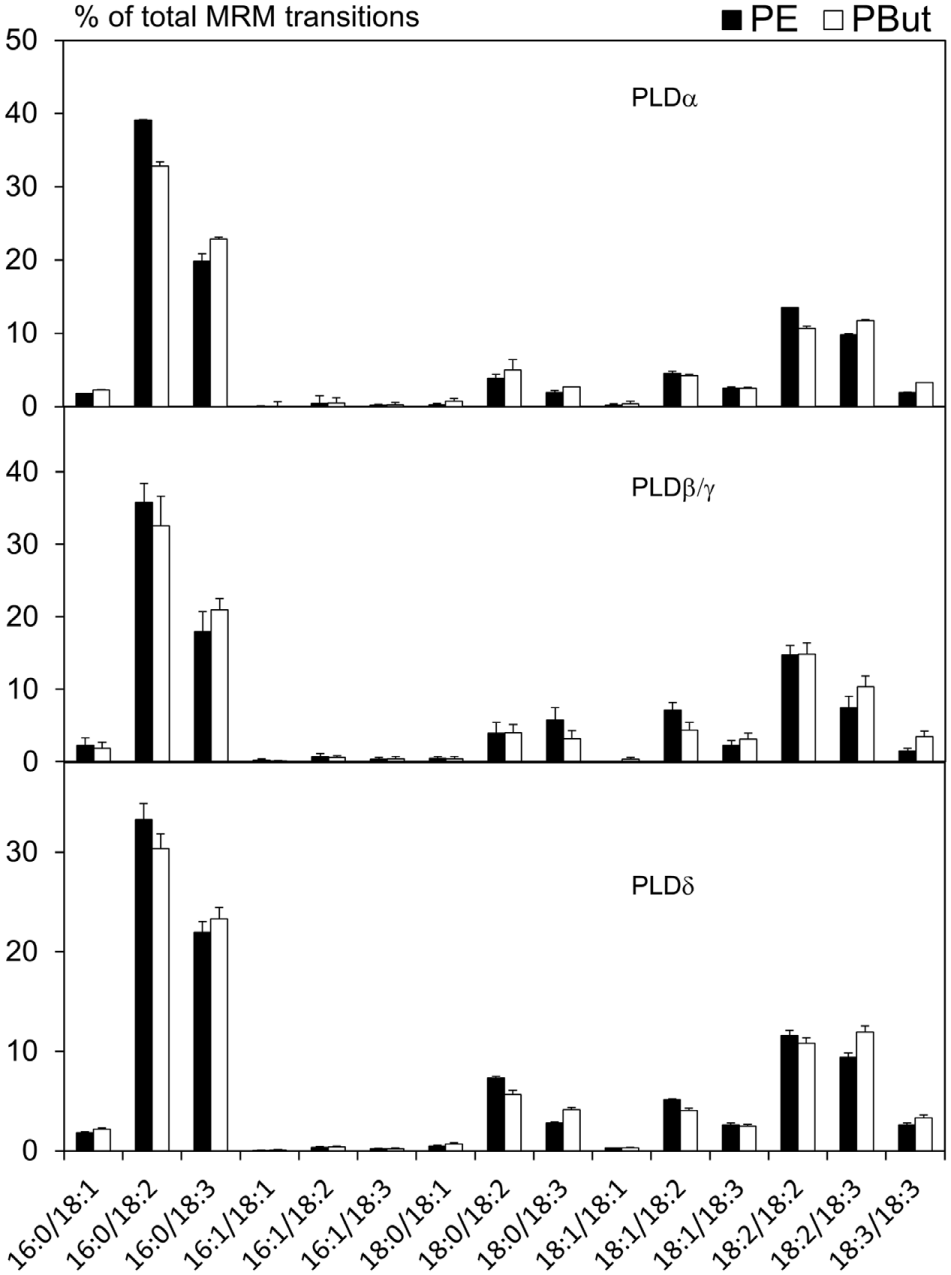
Profiles of PBut produced *in vitro* by Arabidopsis microsomal PLDs. Microsomes were used in an enzymatic assay on lipid vesicles. The substrate used was PE. The reaction assay was defined as α-type, β/γ-type or δ-type. The reactions were performed at 37°C for 20 min in the presence of 0.6% (v/v) *n-*butanol. White bars, substrate; black bars, PBut.

The Arabidopsis genome encodes 12 PLD enzymes that are divided into α, β, γ, δ, ε, and ζ subclasses based on distinct biochemical characteristics [26], [27]. *In vitro*, PLDα and PLDε enzymes are independent of phosphatidylinositol-4,5-bisphosphate (PI(4,5)P_2_) and can act on PC or PE single class micelles in the presence of millimolar concentrations of calcium and SDS. On the contrary, PLDβ and PLDγ enzymes are PI(4,5)P_2_-dependent and can act on micelles composed mainly of PE in the presence of micromolar concentrations of calcium. PLDδ is activated by oleate and requires micromolar concentrations of calcium. PLDζ enzymes are calcium-independent, PI(4,5)P_2_-dependent and can hydrolyze micelles composed of a single lipid class. A series of PLD *in vitro* assays was therefore devised to distinguish between these different activities using lipid vesicles as substrates and microsomal membranes from Arabidopsis cells as sources of enzymes. In the PLDα and PLDδ reaction assays, we used PE and PC separately as substrates. For PLDβ/γ assays, we used vesicles made of PE and PI(4,5)P_2_ (as micelles of PC and PI(4,5)P_2_ are not *in vitro* substrates for PLDβ/γ). All PLD reactions were performed in the presence of 0.6% (v/v) *n-*butanol. The activities detected in PLDα-, PLDβ/γ-, or PLDδ-reaction assays led to PBut products whose molecular species compositions almost perfectly matched the profile of the substrate used, either PE (Figure 5) or PC (not shown). No activity was detected in the PLDζ reaction assay, most likely due to the low level or absence of PLDζ activity in Arabidopsis suspension cells. The results suggest that the enrichment effect observed *in vivo* does not directly arise from the individual PLD activities.

### Arabidopsis PLDs Use PG but not PI as Substrate in vitro

PBut produced by PLD *in vivo* was particularly abundant in 16∶0/18∶2- and 16∶0/18∶3-species. As these are the major species found in PI and PG, we investigated whether the same composition of PBut could be produced *in vitro* by adding either PI or PG to PC:PE (1∶1) micelles in either PLDα or PLDβ/γ reaction assays. While the addition of PI had no effect on PBut profiles (data not shown), the addition of 18∶1/18∶1-PG led to a PG-dose-dependent increase in 18∶1/18∶1-PBut species (Figure S4) showing that PG is a substrate for PLDs.

### Major Glycerophospholipids of Membrane Fractions from Arabidopsis Suspension Cells

**Figure 6:**
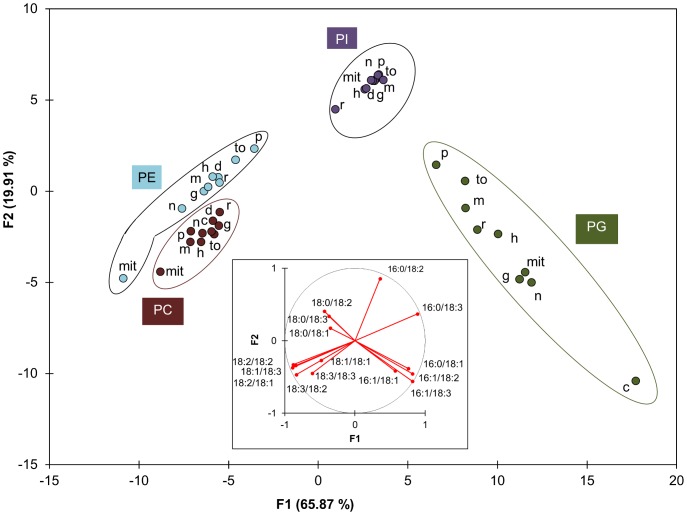
Discriminant analysis of molecular species profiles according to lipid class and membrane fraction. PE, PC, PI and PG profiles were analyzed by MRM mass spectrometry in different membrane fractions. The number of repetitions *n* for each phospholipid class and for each membrane fraction is indicated in Figure S6G. The profiles were used for a discriminant analysis. The score plot represents 86% of the total variability of molecular species profiles. F1/F2 are the 2 principal eigenvalues for this variability. The Variables (phospholipid molecular species)/Factors (F1 and F2) correlations are shown in the loading plot (insert). The score for each profile was calculated. For clarity, only the centroid corresponding to one phospholipid class in a specific membrane is shown. Each class associates a specific phospholipid (PC, PE, PI, PG) with the extract prepared from a sub-cellular membrane fraction. The full image where all scores are presented is in Figure S6E. Abbreviations: mit,mitochondria; n, nucleus; g, Golgi apparatus; m, microsomes; h, homogenate; d, DRM; r, RE; to, tonoplast; p, plasma membrane.

**Figure 7:**
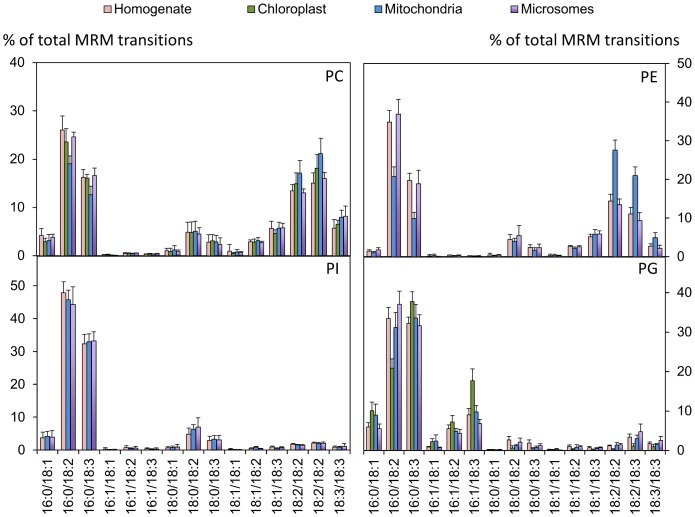
Profiles of PE, PC, PG and PI as analyzed by MRM mass spectrometry in different membrane fractions. *N* = 14, 13, 9 and 4 for homogenate, microsomes, plasma membrane enriched fractions and mitochondria-enriched fractions, respectively. Note that there is no PI or PE in chloroplasts, and that the signals obtained were omitted because they were due to contamination. Diagrams of the molecular species in all analyzed fractions can be found in Figure S6.

Another possible explanation of the specific composition of PBut produced by PLD activity is that it reflects the composition of the membrane where the PLD was located and active in the intact cell. Indeed it is unlikely that all PLD isoforms occur or act homogeneously in all membranes. We therefore separated different membrane fractions of Arabidopsis suspension cells: chloroplasts, mitochondria, nuclear membranes, microsomes, and microsome–derived membranes such as endoplasmic reticulum, Golgi, plasma membrane, tonoplast and detergent-resistant membranes. Enrichment of particular membrane fractions was checked by western blots of corresponding marker proteins (Figure S5). Nuclear fractions were checked by DAPI staining before extracting the membranes (not shown). Figure 6 displays a discriminant analysis (DA) of the molecular species profiles according to lipid class (PE, PG PC and PI) and the membrane fraction. The profiles of the glycerophospholipids in homogenate, microsomes, chloroplasts and mitochondria are shown in figure 7. The profiles of all membrane fractions are displayed in Figure S6.

The DA clearly illustrates that the 4 different glycerophospholipid classes have distinct and separate profiles, whatever membrane is considered. The PI profiles are nearly identical in all fractions, where 16∶0/18∶2- and 16∶0/18∶3-species accounted for more than 75% of the total species. PC is characterized by slight differences between fractions with the mitochondrial PC being the most different. The same is true for PE, but the difference is more pronounced. Indeed for PC and PE it is informative to compare the relative level of the saturated 16∶0/18∶2 and 16∶0/18∶3 species to the diPUFA species. For mitochondrial membranes these ratios were lower than in the homogenate. According to the DA, the molecular species of PG in the different fractions are well dispersed, with the chloroplast PG being isolated. This corresponds to the fact that PG was richer in 16∶1/18∶3- and 16∶0/18∶3-species in chloroplasts than in other fractions. However the level of 16∶1/18∶2- and 16∶1/18∶3-species in the PG profiles obtained was not as high as would be expected based on data from Arabidopsis plants [23]. To verify that no bias had been introduced by the extraction or analysis methods used that might alter the relative proportion of PG molecular species, we used the same MRM method to characterize lipids extracted from Arabidopsis leaves (Figure S7). The PG fatty acid profile from leaves matches published data obtained using different methods. This shows that there are inherent differences in the membrane composition between Arabidopsis suspension cells and leaves.

In no fraction can we observe an enrichment in 16∶0/18∶3- and 16∶0/18∶3-molecular species, relative to the homogenate, comparable to the enrichment seen in PBut relative to PC (at least 1.5-fold for 16∶0/18∶2- and 16∶0/18∶3-species) or PE (nearly 2-fold for 16∶0/18∶3-species) in the bulk lipid extract (see inserts of figure 4). It is therefore not possible to deduce which membrane is the source of the substrate for PLD *in vivo* in this manner.

## Discussion

### MRM is a Reliable Method to Analyze Arabidopsis Glycerophospholipids

Here we used mass spectrometry in the MRM mode to analyze the fatty acid composition of the major glycerophospholipids in Arabidopsis. As far as we know, this is the first time that plant phospholipids have been analyzed in this way. Most mass scan data are obtained using the neutral loss- or precursor of- strategies [28] which indicate the mass, but for glycerophospholipids a single mass can correspond to more than one molecule (Table 1). The MRM strategy has the advantage of giving the exact composition of the glycerophospholipids. The MRM approach was thoroughly tested using phospholipids and enzymes from different sources (i.e., *in vivo* and *in vitro* assays, artificial PBut product vs endogenous substrates, purified cabbage PLD activity vs activity from Arabidopsis extracts, bulk or fractionated membranes from soybean, Arabidopsis plants or suspension cells). MRM is therefore a reliable method for revealing the detail of glycerophospholipid composition in plants.

### Arabidopsis Whole Plant and Suspension Cell Membrane Phospholipid Profiles Differ

The major phospholipids were extracted from 5-day-old cell suspensions or 4-week-old plants and compared. Total lipids from suspension cells had more 16∶0/18∶3- and 16∶0/18∶2-PE species compared to lipids from whole plants. The same was true for PC to a lesser extent. For PG, there was a marked abundance of 16∶1/18∶3- and 16∶0/18∶1-species in membranes from plants compared to cell suspensions. These differences could be due to the lower chloroplast content of suspension cells and/or the different stage of plastid development and hence differences in the relative abundance of each membrane type. As such, these differences highlight the fact that suspension cells should not necessarily be considered as a simplified whole plant system. When we expressed the molecular species obtained in the plant extracts in the C number:unsaturation nomenclature to compare them with previous plant lipid species data recorded in terms of exact *m*/*z* and retention time [25], we found very similar profiles of PE, PC and PG (including the still controversial 36-C PG). For 34-C PG, Burgos et al. [25] hypothesized that 34∶4-PG could result from the introduction of a double bond in the 16 C chain and we indeed detected 18∶3/16∶1-PG species by MRM.

### PBut as an Indicator of PA-producing PLD Activity

To analyze PA specifically due to PLD action, we analyzed the *in vivo* product of PLDs in the presence of *n*-butanol, PBut. This strategy has already been used and is considered valid as long as no technical or biological bias is introduced. When PBut was produced *in vitro* from either PC or PE using purified cabbage PLD and a diethyl ether/buffer reaction mixture, the PBut MRM profile was identical to that of the substrates, showing that no technical bias had artificially exaggerated the presence of certain species from PBut. In parallel we analyzed the PA produced by the PLDs *in vitro* using lipid micelles as substrates (data not shown). The composition was similar to that of PBut, showing that the presence of primary alcohols had no effect on a putative substrate selectivity of the different PLDs assayed.

### SA Alters the Overall Intensity but not the Composition of the PBut Lipid Profile

The profile of PBut was analyzed at different times after the addition of SA, or at 100 min after the addition of different amounts of SA. The PBut profiles did not differ qualitatively between samples, and were characterized by high amounts of 16∶0/18∶2- and 16∶0/18∶3-saturated species compared to diPUFA species. The product of the SA-activated PLDs was not different from the product of the basal activities, but the total amount was increased 2.5 fold. It is this increased level that is therefore likely to be the trigger in the signalling cascade, but it is not known whether this is related to all molecular species or to specific ones.

The fact that plant PLDs produced a variety of PA molecular species is in accordance with data obtained using PLD mutants. For instance, the PA level in the *pldζ2* mutant was lower than in WT plants in response to phosphate deprivation. As there was less of all PA molecular species in the mutant, PLDζ2 probably produces a variety of PA molecular species [29]. A similar conclusion was drawn from the characterization of a *pldε* mutant which contained less PA than WT plants in presence of 2 mM nitrate, although the molecular profile was unaltered [30].

### The in vivo PBut Phospholipid Profile does not Match Profiles of Either PC or PE from Homogenate

In the PLD-catalyzed reaction, the acyl chains of the substrate are unaltered so ‘what goes in is what comes out’. The composition of PLD products can be therefore a key to determine *in vivo* PLD substrates. For example, Arisz et al. [22] showed that the stress-activated PLD in Chlamydomonas used PE as a substrate, because the analyzed PBut contained an 18∶1/18∶1-species that is only found in PE from Chlamydomonas. However when we compared the composition of PBut with those of the major glycerophospholipids in Arabidopsis, strikingly, the PBut profile did not match that of either the PC or the PE present in the bulk suspension cell lipid extract. Different explanations can be proposed for this: (i) the PLD selected substrate lipids with a particular subset of fatty acids; (ii) the PLDs could use either PI or PG as well as PC and PE; (iii) molecular species were unevenly distributed over cellular membranes [22]. We tested these 3 possibilities.

It was hypothesized that PLDs would choose specific molecular species within a certain class of glycerophospholipid. For instance the *in vivo* PBut profile would fit the hypothesis of PC being the substrate, and PLD preferentially accepting the 16∶0/18∶2- and 16∶0/18∶3-PC molecular species. However, none of the PLDs present in Arabidopsis suspension cells were selective *in vitro* (Figure 5).

### PC, PE and PG are Substrates of Arabidopsis PLD in vitro but not Necessarily in vivo

The observed PBut profile could arise from PLD using either PI or PG in addition to PC which would explain the abundance of the 16∶0/18∶2- and 16∶0/18∶3-molecular species that are predominant in PI and PG in suspension cells. We showed that PI was not a substrate for Arabidopsis PLDs *in vitro* in keeping with published data [26], [27]. We also showed that when PG was added to PE:PC micelles, it was used as a substrate. This result is consistent with experiments showing PG as a substrate of recombinant PLDα1, PLDβ1, PLDγ1 [27], and PLDε [30] but not of PLDζ1 [26]. However PG is characterized by the presence of 16∶1 acyl chains which were not detected in PBut, so this suggests that PG is not an *in vivo* PLD substrate in control or SA-treated cells.

The difference between the profiles of PBut and those of the putative PLD substrates could result from a heterogeneous distribution of substrates in cell membranes. We analyzed PC, PE, PI and PG from different membrane fractions but the PBut profile did not match those of either PC or PE in any individual fraction.

This might suggest that PLD acts on membrane microdomains of a particular composition. The PBut composition did not match that of DRMs prepared from microsomes, essentially a large pool derived from plasma membranes, although DRMs also derive from Golgi [31]. Other kinds of microdomains may exist, such as the non-DRM fraction of the membrane. It is also highly probable that the different glycerophospholipid classes are asymmetrically distributed between the two membrane layers of the plasma membrane [32], due to the presence of aminophospholipid translocase, an enzyme which can pump PE from the outer membrane leaflet to the inner one [33]. Since PLDs are likely to be active on the cytoplasmic face of the membrane, this asymmetrical distribution of substrate could be a factor in determining the composition of end-products. Finally, the observed enrichment in 16∶0/18∶2- and 16∶0/18∶3-species in PBut can also be seen as a relative depletion in the diPUFA species. Previous studies on membranes have reported that the degree of unsaturation affects important bilayer properties such as molecular order [34], acyl chain packing [35] or membrane area per molecule [36]. The presence of unsaturated bonds in *sn-1* and/or *sn-2* acyl chains can indeed affect how an extrinsic protein interacts with a membrane, as shown with protein kinase C [37]. PLDs are also sensitive to the presence of diPUFA phospholipids in the bilayer that can segregate from other membrane components such as sterols [38], so this might explain why some molecular species are less hydrolyzed than others. The action of PLDs on artificial membranes composed of different ratio of diPUFA vs saturated lipids in the presence or absence sterols might be a way of testing this hypothesis.

## Materials and Methods

### Cell Culture

*Arabidopsis thaliana* Col-0 suspension cells were cultivated as in [7]. Experiments were performed on 5-d-old cultures.

### Lipid Extraction

Cells (7 mL of cell suspension, approximately 1 g wet weight) were pelleted and the media removed. Standard lipids were added before cell lipids were extracted by the addition of 10 mL hot methanol that stopped all biological reactions. After vortexing, 10 mL of chloroform/33% (v/v) HCl (100∶1.5, v/v) was added. A two-phase system was produced by the addition of 3 mL of 0.9% (w/v) NaCl. The apolar phase was recovered, dried under a nitrogen stream and resuspended in 400 µL chloroform supplemented with 0.02% (v/v) butylated hydroxytoluene (BHT). Samples were kept at −20°C before analysis.

### Mass Spectrometry

The HPLC separation was performed using an Agilent 1100 HPLC system using a 250 mm×4 mm (length × internal diameter) 5 µm Lichrospher silica column, at 65°C. The mobile phases consisted of hexane/isopropanol/water (628∶348∶24, v/v) supplemented with 10 mg/L ammonium formiate (A) and isopropanol/water (850∶146, v/v) supplemented with 10 mg/L ammonium formiate (B). The injection volume was 5 µL. The percentage of B was increased linearly from 0% to 40% in 45 min and then to 100% in 3 min. This elution sequence was followed by an equilibration for 2 min with 100% B before returning to 100% A. Finally the column was equilibrated for 8 min, leading to a total runtime of 60 min. The flow rate of the mobile phase was 300 µL/min. The distinct glycerophospholipid classes were eluted successively as a function of the polar head group.

Phospholipids were detected and the structure ascertained by CID in the tandem mass spectrometer (QTrap2000, ABSciex) used in the negative (capillary voltage −4500V) or positive mode (capillary voltage +5000V) depending on the nature of the fragmentation of the glycerophospholipid head group. Precursor ion and neutral loss were used as detection mode depending on the particular glycerophospholipid. The elution of phospholipids triggered an Enhanced Resolution (ER) mass spectrum of the parent phospholipid ions. The triggering was piloted by a preset of the Informative Dependent Acquisition (IDA) software module of Analyst 1.4.2 software. The quantification of identified phospholipid molecular species was performed in the Multiple Reaction Monitoring (MRM) mode. MS2 scan was used to obtain the parent phospholipid fatty acid composition by CID.

### Deisotopization

It is necessary to take into account the isotopic distribution of C in phospholipid molecules. We considered ^13^C to occur at 1.1% of the frequency of ^12^C. For each MRM transition, we calculated a correction coefficient considering that the monitored transition is in part due to an isotopic overlap. For instance, the 16∶1/18∶3-PC will be monitored with the MRM experiments 798.5 → 277.2 and 798.5 → 253.2. Yet if the molecule incorporated two ^13^C, it will lead to a molecule of *m/z* 800.5. If the two ^13^C are incorporated in the 16∶1 fatty acid, it will be monitored with the transition 800.5 → 255.2 (overlapping with the signal due to 16∶0/18∶3-PC). If the two ^13^C are incorporated in the 18∶3 fatty acid, the molecule will then be monitored by the 800.5 → 279.2 transition (overlapping with the signal due to 16∶1/18∶2-PC). If the two ^13^C are incorporated in the glycerophosphocholine fragment of the molecule, it will be monitored as 800.5 → 277.2 and 800.5 → 253.2 MRM transitions (overlapping with the signal due to 16∶0/18∶3-PC and 16∶1/18∶3-PC, respectively). Therefore the signal intensities obtained for 800.5 → 255.2, 800.5 → 279.2, 800.5 → 277.2 and 800.5 → 253.2 MRM transitions, to be attributed to 16∶0/18∶3-PC, 16∶1/18∶2-PC, 16∶0/18∶3-PC and 16∶1/18∶3-PC, respectively, have to be corrected for the isotopic overlap with 16∶1/18∶3-PC. The correction factor was calculated for each transition as
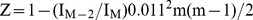
where m is the carbon number of the part of the molecule where the two ^13^C are present (fatty acids or glycerol-phospho-head group) in the molecule at the lowest molecular weight to lead to an isotopic contamination of the signal at M molecular weight, I_M−2_ and I_M_ are the peak intensities of MRM transitions at molecular weight (M−2) and M, respectively [39]. Such correction was done when necessary for all MRM transitions, for all glycerophospholipids, from the lowest to highest molecular weights. The Excel macro developed is in Table S1.

### Preparation of Arabidopsis Glycerophospholipids for in vitro PLD Activity

Lipids were extracted as described above from approximately 100 mL of Arabidopsis suspension culture. PI, PC and PE were separated by high-pressure liquid chromatography (HPLC) on a column packed with silicic acid (10 µm, µPorasil) according to [40]. PE was further separated from phosphatidylglycerol (PG) by HPLC in an isocratic mode with isopropanol/hexane/H_2_O (34.8∶62.8∶2.4, v/v). The purified fractions of PC and PE were quantified by mineralization of the diester phosphate and phosphate measurement [41].

### Preparation of PBut by in vitro Cabbage PLD Activity

Reactions were performed in a biphasic reaction system as described in [42] using cabbage PLD (Sigma-Aldrich). PE (347 µg) or PC (674 µg) were resuspended in diethyl ether and incubated in 300 mM sodium acetate buffer pH 5.5, containing 120 mM CaCl_2_ in the presence of 1.2% (v/v) *n-*butanol.

### In vitro Activity of Arabidopsis PLDs

For the PLDα-type reaction assay, microsomal proteins were incubated in 50 mM MES pH 6.5, 0.5 mM SDS, 60 mM CaCl_2_, 1.5% Triton X-100 (modified from [43]). For the other reaction assays, the buffer was 100 mM Tris-HCl pH 7, 80 mM KCl. This reaction buffer was supplemented with 0.5 mM MgCl_2_, 50 µM CaCl_2_ and 0.1% Triton X-100 for PLDβ/γ-type reaction assay (modified from [44]); it was supplemented with 2 mM MgCl_2_, 100 µM CaCl_2_ and 0.01% Triton X-100 for the PLDδ-type reaction assay (modified from [26]); and it was supplemented with 2 mM EGTA, 2 mM EDTA, 0.1% Triton X-100 for the PLDζ-type reaction assay (modified from [26]). For PLDα , reactions were performed in the presence of 60 µg of either PC or PE. PLDδ reaction assays comprised 30 µg PC or PE with 0.12‰ (v/v) oleate. For PLDζ, reaction assays were performed with 36 µg PC and 3.6 µg phosphatidylinositol-4,5-bisphosphate (PIP2); and for PLDβ/γ, 30 µg PE and 3.6 µg PIP2 were used. The reactions were performed in 120 µL in the presence of 0.6% (v/v) *n-*butanol at 37°C for the PLDα-type assay and 30°C for the other PLD assays. Reactions were stopped by the addition of 1 ml of chloroform/methanol (2∶1, v/v). Standard phospholipids were added and a two-phase system was created by the addition of 100 µL of 1 M KCl. The organic phase was air-dried, and the residue dissolved in 400 µL of chloroform supplemented with 0.02% (v/v) BHT.

### Membrane Isolation from A. thaliana Suspension Cells

Cells were ground in homogenization buffer (1 mL g^−1^ fresh weight) containing 100 mM MOPS-KOH (pH 7.5), 600 mM sucrose, 4 mM EDTA, 0.1 mM PMSF, 5 mM DTT, 5 mM ascorbic acid, 0.05% (w/v) cysteine, 0.6% (w/v) PVP K25 and 0.2% (w/v) BSA. From this homogenate, nuclei were isolated as in [45] and [46]. Chloroplasts were isolated according to [47]. Mitochondria were obtained according to [48]. From microsomes, we isolated either plasma membranes, or detergent-resistant membranes, or endoplasmic reticulum and Golgi membranes or tonoplasts [31], [46], [49]–[51]. A schematic representation of the cell fractionation procedure leading to the different membranes is given in Figure S8. Lipids were extracted according to [52].

### Western Blot Analysis

Ten µg of membrane proteins solubilized in a Laemmli denaturation buffer were separated by SDS-PAGE and transferred to nitrocellulose membranes. The primary antibodies raised against H^+^-ATPase isoform 2 (AHA2) [53], AtMemb11 [54], AOX [55], TIC40 (Agrisera), SMT1 [56] and epsilon subunit of tonoplast H^+^-ATPase (Agrisera) were used for detection of plasma membrane, Golgi, mitochondria and chloroplast, endoplasmic reticulum and tonoplast, respectively.

### Statistical Analysis

Discriminant analysis (DA) of 225 phospholipid molecular species (Table A of Figure S6G) was performed using XLSTAT 2011.4.04 (Addinsoft) software. Within-class covariance matrices are assumed to be different as indicated by the Box test (Fisher’s F asymptotic approximation) and Kullback’s test.

## Supporting Information

Figure S1 **Representative MRM experiment.** (A) Sum of the signals of all MRM transitions analyzed. The peaks corresponding to the elution of PE, PC and PI can be visualized. (B) Each MRM transition for one glycerophospholipid class can be visualized separately, as shown with PI transitions. PI is composed mainly of two molecular species (16∶0/18∶2-PI and 16∶0/18∶3-PI) that are analyzed through four MRM transitions. These four MRM transitions are the four biggest ones while the other 23 MRM transitions (see Table 1 the list of 27 transitions for each glycerophospholipid class) give very low signals. (C) Signal intensities are associated with each MRM transition for one lipid class, leading to the MRM spectrum for this lipid. PI spectrum is displayed. This spectrum is calculated within the time period corresponding to PI elution, therefore the data are not contaminated by isobars that would elute at different time period.
(PPT)

Figure S2 **Analysis of PBut produced by the** ***in vitro*** **action of cabbage PLD on Arabidopsis PC or PE.** The reaction was performed at 30°C for 4 h in a two-phase system composed of diethylether and sodium acetate buffer in the presence of 1.2% (v/v) *n-*butanol. Lipids were analyzed by mass spectrometry in the MRM mode, searching for the transitions listed in Table 1.
(TIFF)

Figure S3 **Profiles of PBut as a function of time and/or SA concentration.** Profiles of PBut extracted 80 min after cells were treated with 125, 500, 750 and 1000 µM SA (A). Profiles of PBut extracted 60, 120 or 240 min after cells were treated with 750 µM SA (B). PE and PC profiles of the corresponding bulk lipid extracts are shown. Lipids were analyzed by mass spectrometry in the MRM mode by searching for the transitions listed in Table 1. In (B), transitions for the minor 16∶1/18∶2- and 16∶1/18∶3-species were not analyzed.
(PPT)

Figure S4 **Profiles of PBut produced** ***in vitro*** **by Arabidopsis microsomal PLDs in the presence of di18∶1-PG.** Microsomes were used in an enzymatic assay on lipid vesicles composed of equal amounts of PC and PE, and in the presence of increasing quantities of PG. The reaction assay was defined as α-type or β/γ-type. PC:PE:PG (1∶1∶0), black bars; PC:PE:PG (1∶1∶0.25), grey bars; PC:PE:PG (1∶1∶1), white bars. Lipids were analyzed by mass spectrometry in the MRM mode by searching for the transitions listed in Table 1.
(PPTX)

Figure S5 **Purity of the membrane fractions assessed by western blotting.** The quality of fractions enriched in plasma membrane, reticulum, Golgi, vacuole, mitochondria and chloroplast membranes was assessed using antibodies directed against AHA2, SMT1, Atmemb11, V-H^+^ ATPaseAOX and Tic40, respectively.
(PPTX)

Figure S6 **Glycerophospholipid composition in different membrane fractions.** Profiles of PC (A), PE (B), PI (C) and PG (D) as analyzed by MRM mass spectrometry in the different membrane fractions. Lipids were analyzed by mass spectrometry in the MRM mode, searching for the transitions listed in Table 1. (E) Discriminant Analysis of the molecular species according to the lipid class and membrane fractions. (F) The score plot represents 86% of the total variability of molecular species profiles. F1/F2 are the 2 principal eigenvalues for this variability. Variables (phospholipid molecular species)/Factors (F1 and F2) correlations are shown in the loading plot. Each dot in (E) represents the score of a separate profile which comprises the measurements for 15 molecular species indicated in Table B of Figure S6G. The square symbols represent the centroid of the 39 classes indicated in Figure S6G. Each class associates a specific phospholipid (PC, PE, PI, PG) with the extract prepared from a sub-cellular membrane fraction. (G) Tables A (number of repetitions *n* for each phospholipid class and for each membrane fraction, leading to 225 observations) and B (mean values and standard deviation of the 225 observations for each 15 molecular species).
(PPT)

Figure S7 **Profiles of PE, PC, PI and PG as analysed by MRM mass spectrometry in Arabidopsis leaves.** Lipids were analyzed by mass spectrometry in the MRM mode by searching for the transitions listed in Table 1. The profiles of leaf lipids are shown side by side with that of suspension cell lipids.
(PPTX)

Figure S8 **Cell fractionation protocol.**
(PPT)

Table S1 **Excel Macro to correct for the isotopic contamination of MRM transitions.**
(XLS)
