# Supplementary figures and images for: Acyl Chains of Phospholipase D Transphosphatidylation Products in Arabidopsis Cells: A Study Using Multiple Reaction Monitoring Mass Spectrometry
Source: PLoS One. 2012 Jul 25;7(7):e41985. doi: 10.1371/journal.pone.0041985 (PMC3405027; doi:10.1371/journal.pone.0041985)

## Slide 1
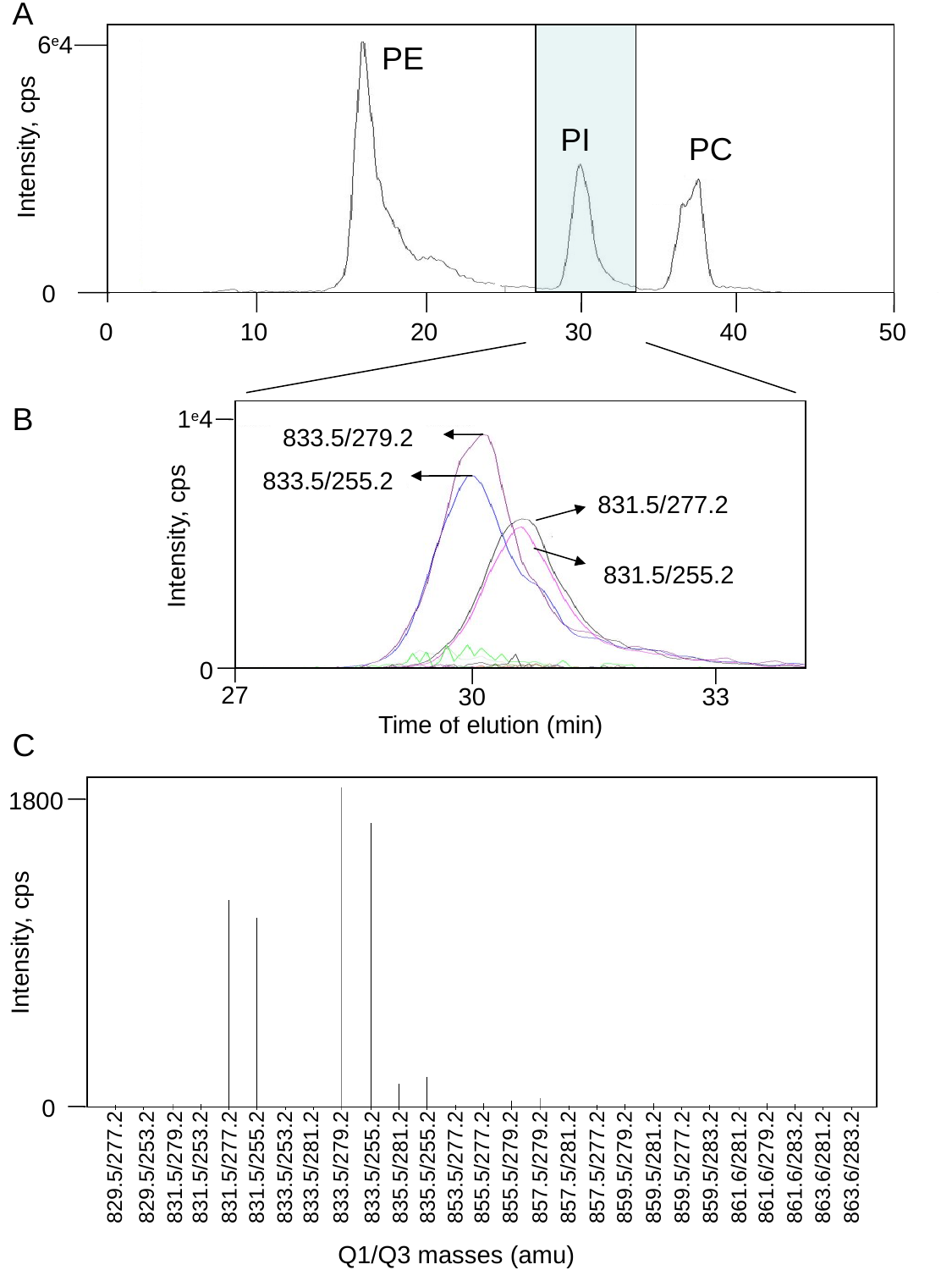

A
6e4
PE
PI
PC
Intensity, cps
0
0
10
20
30
40
50
B
1e4
833.5/279.2
833.5/255.2
831.5/277.2
Intensity, cps
831.5/255.2
0
27
30
33
Time of elution (min)
C
1800
Intensity, cps
0
829.5/277.2
829.5/253.2
831.5/279.2
831.5/253.2
831.5/277.2
831.5/255.2
833.5/253.2
833.5/281.2
833.5/279.2
833.5/255.2
835.5/281.2
835.5/255.2
853.5/277.2
855.5/277.2
855.5/279.2
857.5/279.2
857.5/281.2
857.5/277.2
859.5/279.2
859.5/281.2
859.5/277.2
859.5/283.2
861.6/281.2
861.6/279.2
861.6/283.2
863.6/281.2
863.6/283.2
Q1/Q3 masses (amu)

Supplement: Figure S1 — Representative MRM experiment. (A) Sum of the signals of all MRM transitions analyzed. The peaks corresponding to the elution of PE, PC and PI can be visualized. (B) Each MRM transition for one glycerophospholipid class can be visualized separately, as shown with PI transitions. PI is composed mainly of two molecular species (16∶0/18∶2-PI and 16∶0/18∶3-PI) that are analyzed through four MRM transitions. These four MRM transitions are the four biggest ones while the other 23 MRM transitions (see Table 1 the list of 27 transitions for each glycerophospholipid class) give very low signals. (C) Signal intensities are associated with each MRM transition for one lipid class, leading to the MRM spectrum for this lipid. PI spectrum is displayed. This spectrum is calculated within the time period corresponding to PI elution, therefore the data are not contaminated by isobars that would elute at different time period. (PPT) [file pone.0041985.s001.ppt]
