# Supplementary material for: Acyl Chains of Phospholipase D Transphosphatidylation Products in Arabidopsis Cells: A Study Using Multiple Reaction Monitoring Mass Spectrometry
Source: PLoS One. 2012 Jul 25;7(7):e41985. doi: 10.1371/journal.pone.0041985 (PMC3405027; doi:10.1371/journal.pone.0041985)
Supplement: Figure S3 — Profiles of PBut as a function of time and/or SA concentration. Profiles of PBut extracted 80 min after cells were treated with 125, 500, 750 and 1000 µM SA (A). Profiles of PBut extracted 60, 120 or 240 min after cells were treated with 750 µM SA (B). PE and PC profiles of the corresponding bulk lipid extracts are shown. Lipids were analyzed by mass spectrometry in the MRM mode by searching for the transitions listed in Table 1. In (B), transitions for the minor 16∶1/18∶2- and 16∶1/18∶3-species were not analyzed. (PPT) [file pone.0041985.s003.ppt]

## Slide 1
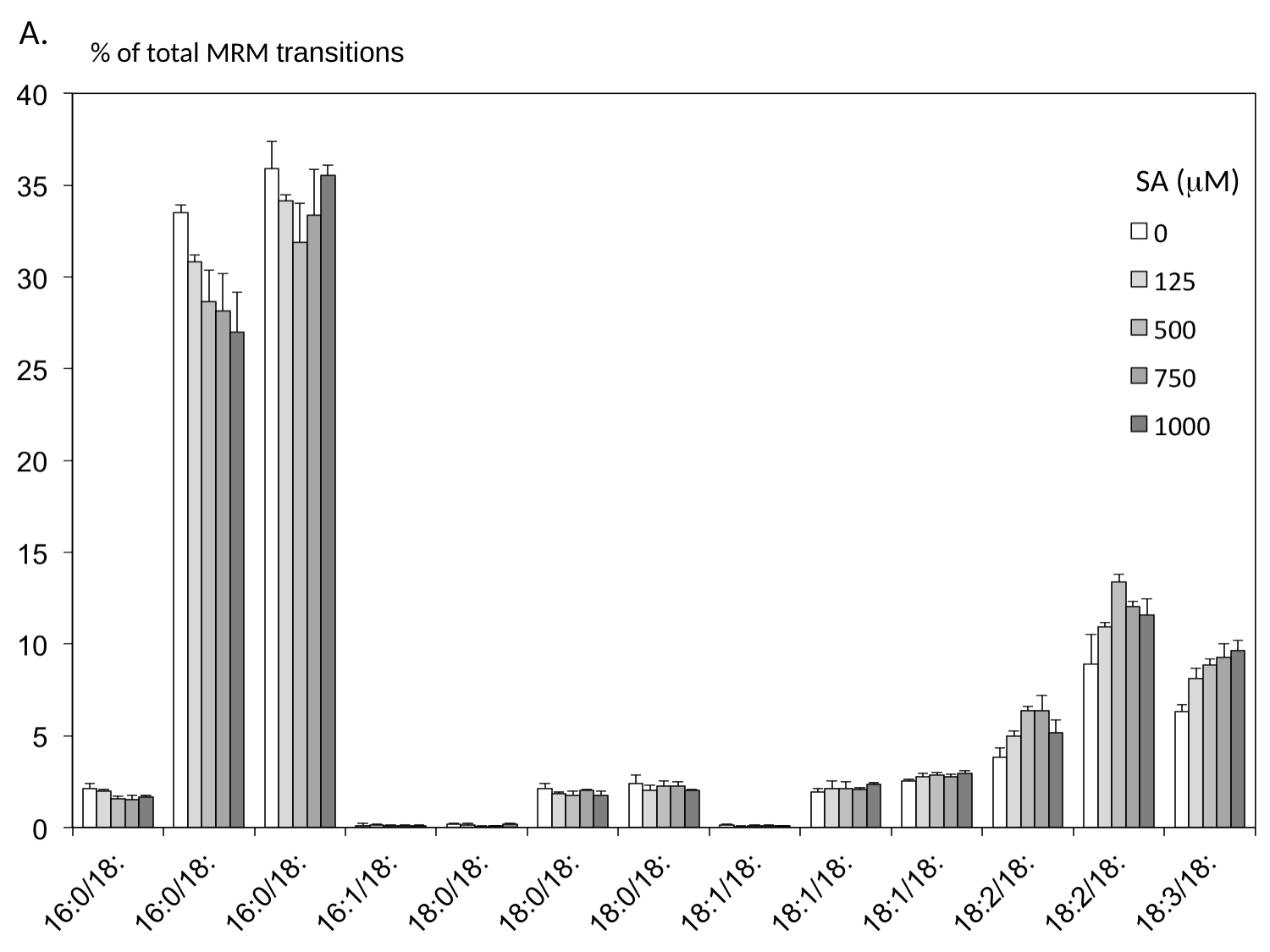

A.
% of total MRM transitions
SA (μM)

## Slide 2
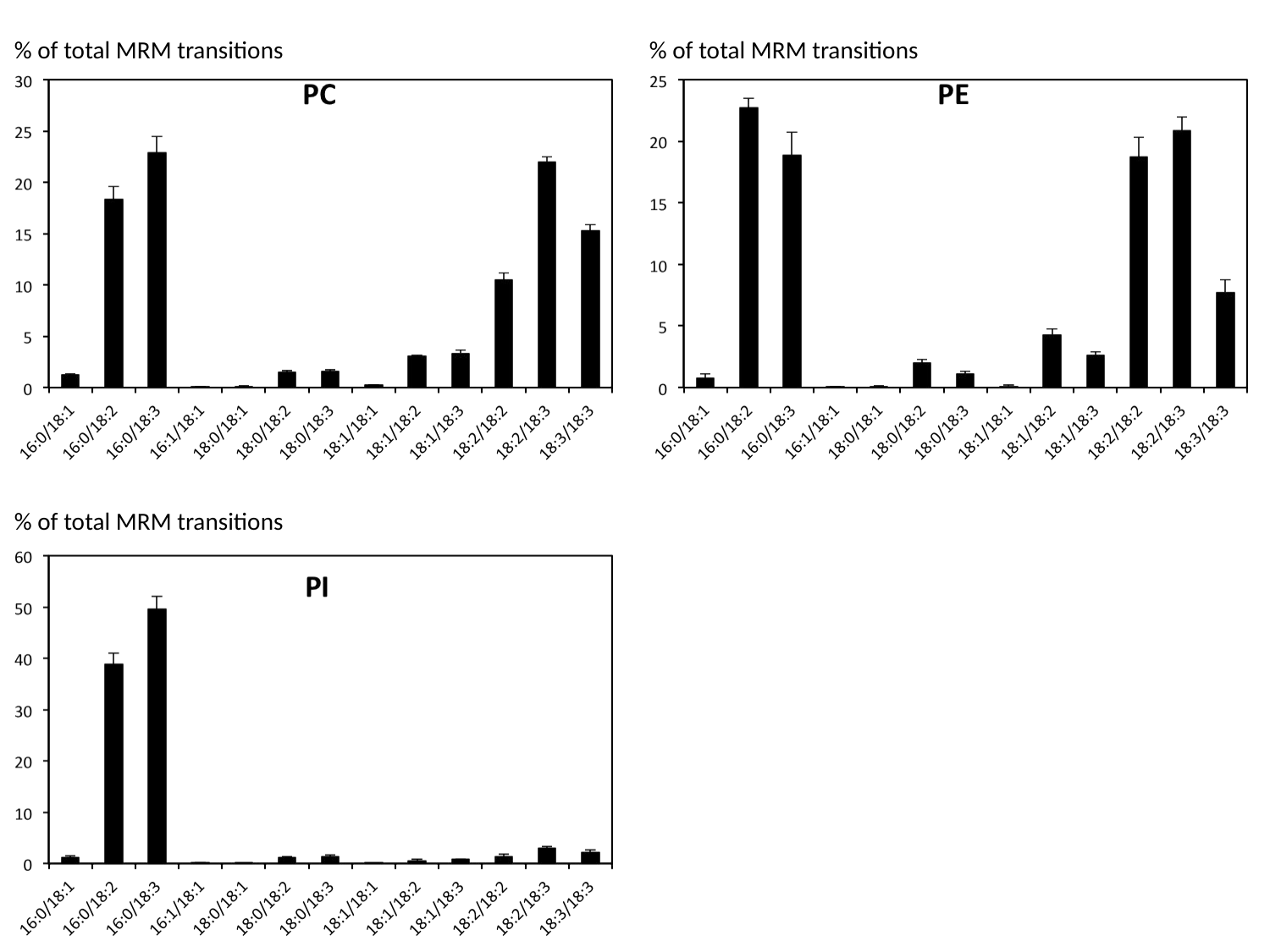

% of total MRM transitions
% of total MRM transitions
% of total MRM transitions

## Slide 3
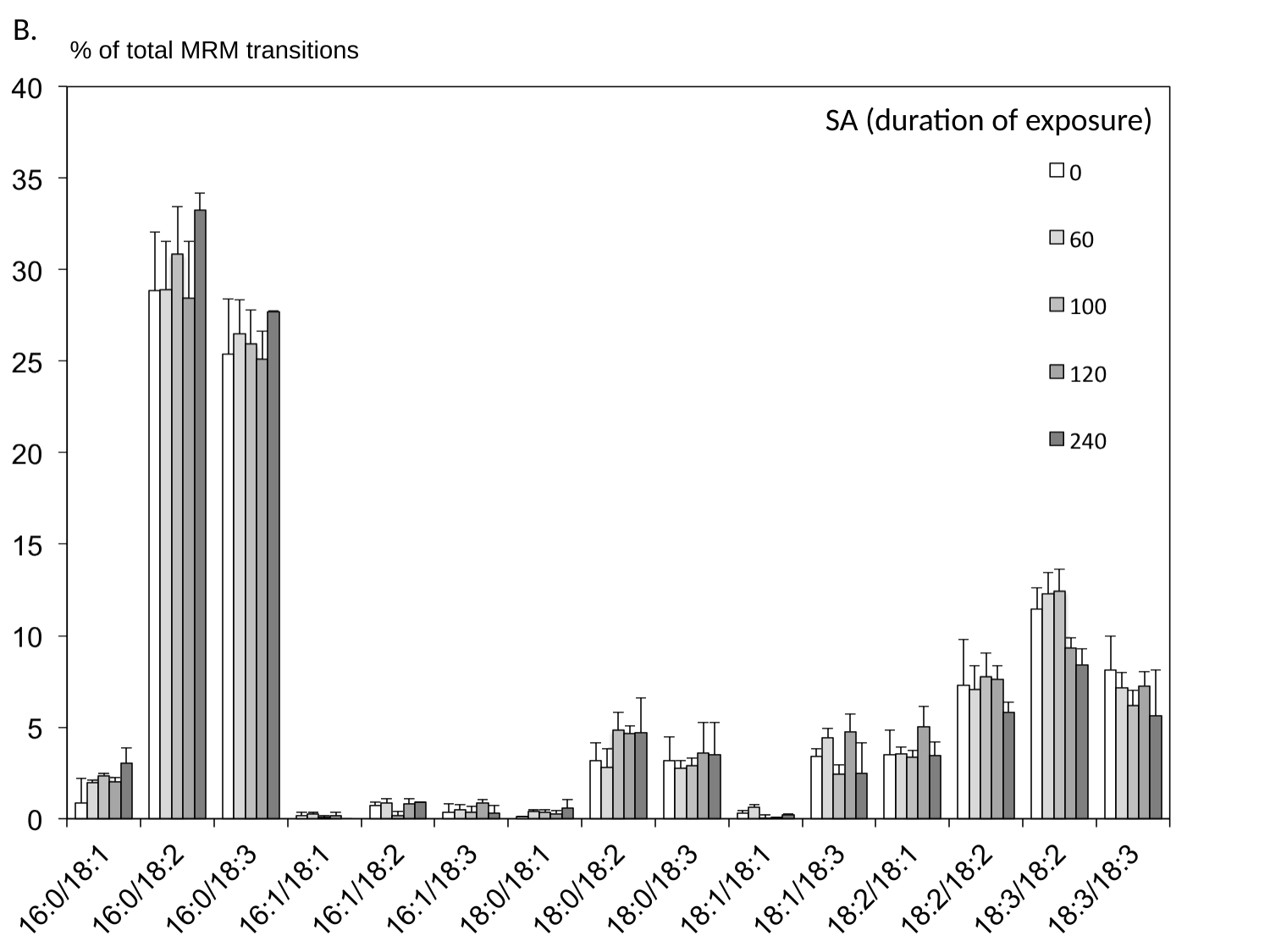

B.
% of total MRM transitions
SA (duration of exposure)

## Slide 4
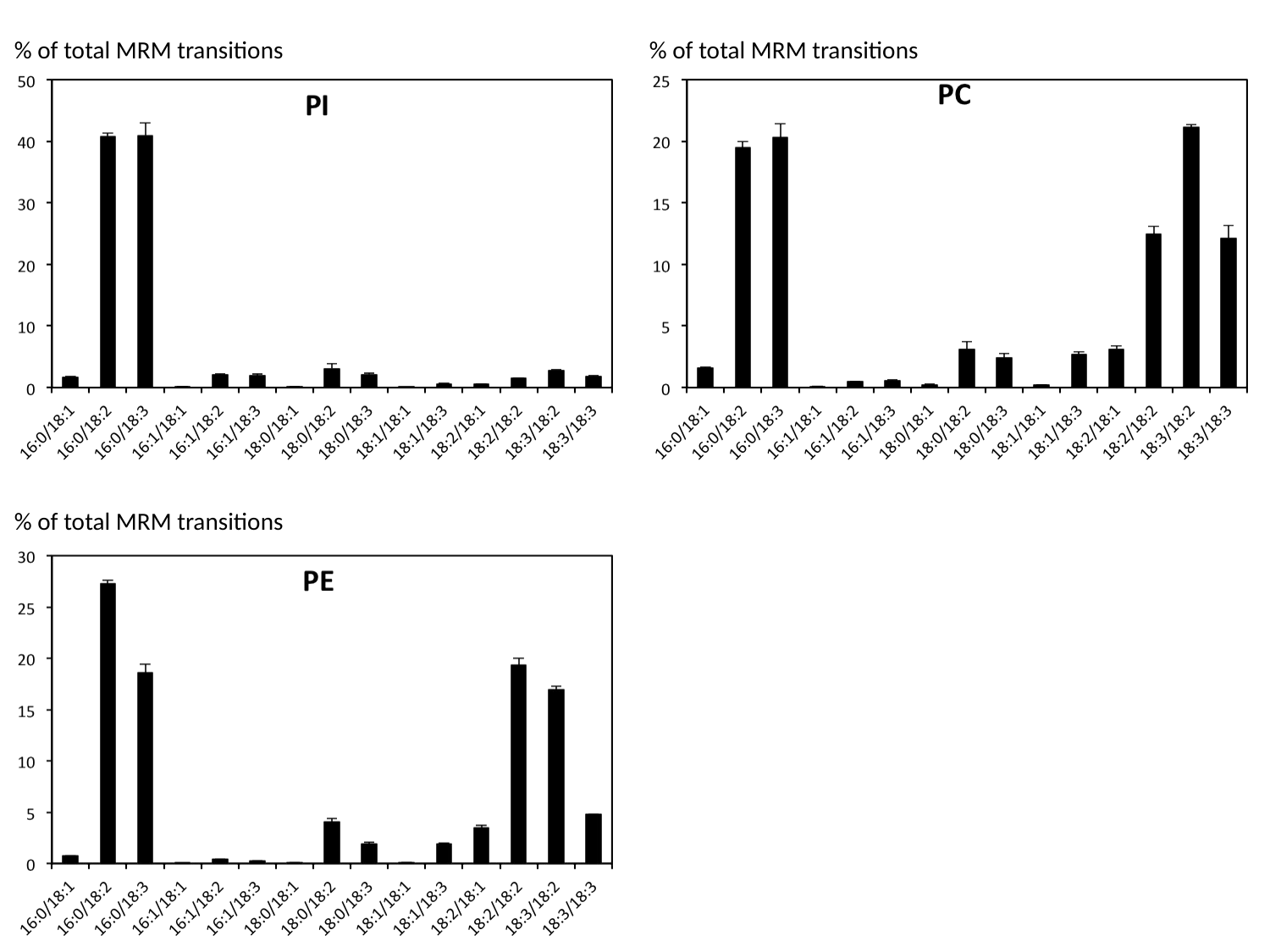

% of total MRM transitions
% of total MRM transitions
% of total MRM transitions
