# Supplementary material for: Acyl Chains of Phospholipase D Transphosphatidylation Products in Arabidopsis Cells: A Study Using Multiple Reaction Monitoring Mass Spectrometry
Source: PLoS One. 2012 Jul 25;7(7):e41985. doi: 10.1371/journal.pone.0041985 (PMC3405027; doi:10.1371/journal.pone.0041985)
Supplement: Figure S4 — Profiles of PBut produced in vitro by Arabidopsis microsomal PLDs in the presence of di18∶1-PG. Microsomes were used in an enzymatic assay on lipid vesicles composed of equal amounts of PC and PE, and in the presence of increasing quantities of PG. The reaction assay was defined as α-type or β/γ-type. PC:PE:PG (1∶1∶0), black bars; PC:PE:PG (1∶1∶0.25), grey bars; PC:PE:PG (1∶1∶1), white bars. Lipids were analyzed by mass spectrometry in the MRM mode by searching for the transitions listed in Table 1. (PPTX) [file pone.0041985.s004.pptx]

## Slide 1
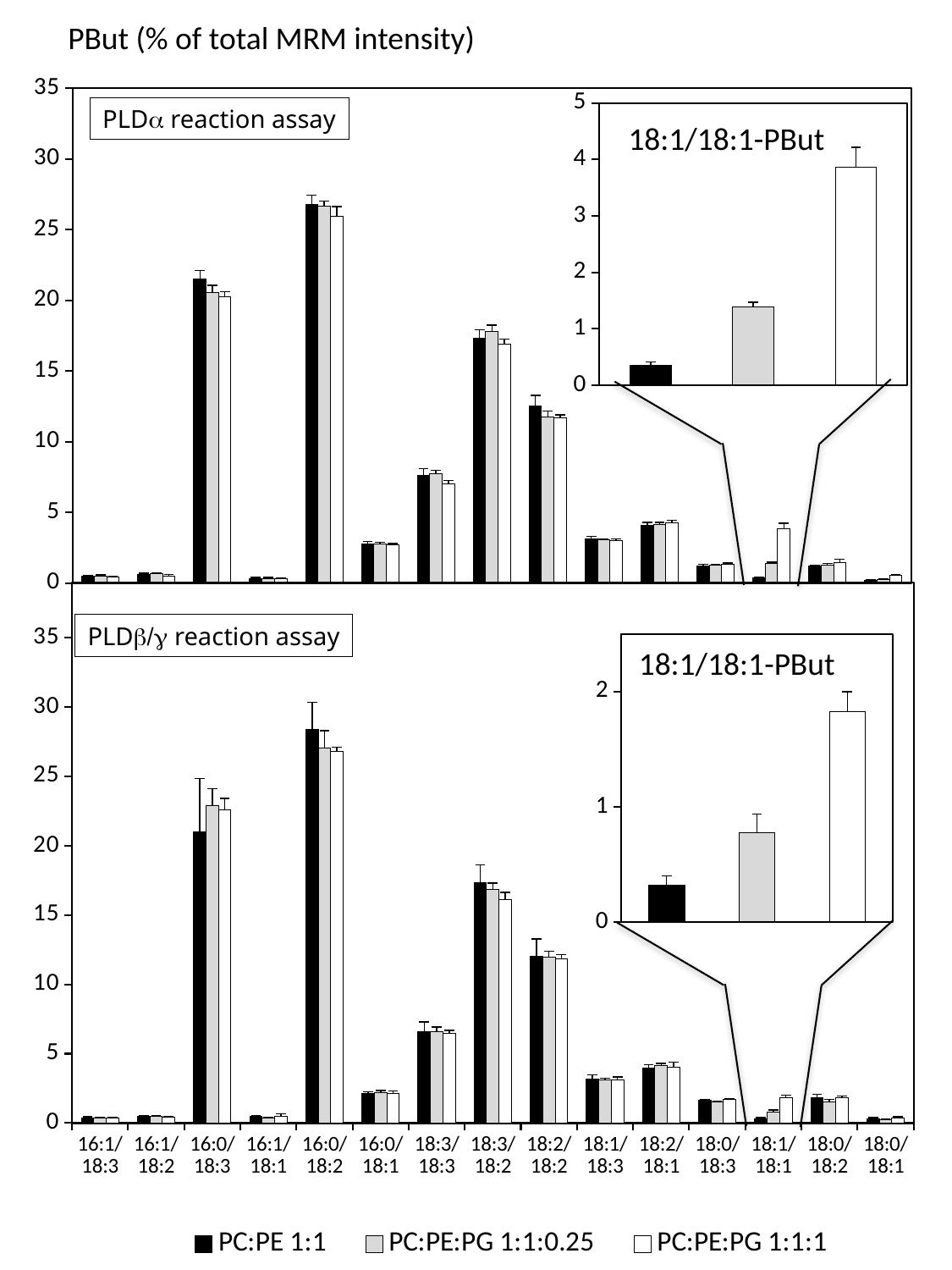

### Chart
| Category | PC:PE 1:1 | PC:PE:PG 1:1:0.25 | PC:PE:PG 1:1:1 |
|---|---|---|---|
| 16:1/18:3 | 0.478881546330318 | 0.464603368700208 | 0.433890370891924 |
| 16:1/18:2 | 0.628800326560324 | 0.641023281996848 | 0.50245109612384 |
| 16:0/18:3 | 21.50132364169391 | 20.55491283242938 | 20.23634023230791 |
| 16:1/18:1 | 0.29615099115988 | 0.319884254656583 | 0.299707150801797 |
| 16:0/18:2 | 26.78361630478656 | 26.64987444100785 | 25.94549233035235 |
| 16:0/18:1 | 2.731844661784791 | 2.74949114269277 | 2.686518888616724 |
| 18:3/18:3 | 7.587175399948308 | 7.716188279928113 | 6.997539431637587 |
| 18:3/18:2 | 17.3349672649508 | 17.76363197558872 | 16.87929045890388 |
| 18:2/18:2 | 12.545746805118 | 11.75645173288043 | 11.69789172281479 |
| 18:1/18:3 | 3.126848749796955 | 3.062333475734691 | 2.967090729375126 |
| 18:2/18:1 | 4.098859439242656 | 4.160193573494227 | 4.23375865181414 |
| 18:0/18:3 | 1.172370875525202 | 1.241756257643758 | 1.293902190282933 |
| 18:1/18:1 | 0.356527052570123 | 1.393164876005055 | 3.861262352924133 |
| 18:0/18:2 | 1.175842216621717 | 1.267877307761043 | 1.450093667313762 |
| 18:0/18:1 | 0.181044723910453 | 0.258613199480301 | 0.514770725838987 |PBut (% of total MRM intensity)
### Chart
| Category | |
|---|---|PLDa reaction assay
18:1/18:1-PBut
### Chart
| Category | PC:PE 1:1 | PC:PE:PG 1:1:0.25 | PC:PE:PG 1:1:1 |
|---|---|---|---|
| 16:1/18:3 | 0.353732336889712 | 0.355348075828619 | 0.332764406453273 |
| 16:1/18:2 | 0.478005094398271 | 0.457458754318343 | 0.419509342801025 |
| 16:0/18:3 | 21.00333219087455 | 22.90489927949091 | 22.59567800847457 |
| 16:1/18:1 | 0.460787175938317 | 0.352699754806581 | 0.471116884145962 |
| 16:0/18:2 | 28.36643019855529 | 27.02778404565062 | 26.82618911607243 |
| 16:0/18:1 | 2.106016304768707 | 2.196507862374402 | 2.124417070714197 |
| 18:3/18:3 | 6.617312871353974 | 6.613549642551915 | 6.453188999331687 |
| 18:3/18:2 | 17.3576544813269 | 16.85915189963625 | 16.12562212955767 |
| 18:2/18:2 | 12.0309766036906 | 11.97373822060142 | 11.84641166714501 |
| 18:1/18:3 | 3.164535340857407 | 3.126085910447498 | 3.116729265833661 |
| 18:2/18:1 | 3.973702593109782 | 4.12981102802375 | 4.028684220648866 |
| 18:0/18:3 | 1.646153412466526 | 1.50965607094369 | 1.673174706523708 |
| 18:1/18:1 | 0.317146596592411 | 0.771869169659978 | 1.826511927769973 |
| 18:0/18:2 | 1.839406784231985 | 1.497405152756842 | 1.800799699410715 |
| 18:0/18:1 | 0.28480801494558 | 0.224035132909189 | 0.359202555117262 |
### Chart
| Category | 18:1/18:1 |
|---|---|PLDb/g reaction assay
18:1/18:1-PBut
