# Supplementary material for: Acyl Chains of Phospholipase D Transphosphatidylation Products in Arabidopsis Cells: A Study Using Multiple Reaction Monitoring Mass Spectrometry
Source: PLoS One. 2012 Jul 25;7(7):e41985. doi: 10.1371/journal.pone.0041985 (PMC3405027; doi:10.1371/journal.pone.0041985)
Supplement: Figure S5 — Purity of the membrane fractions assessed by western blotting. The quality of fractions enriched in plasma membrane, reticulum, Golgi, vacuole, mitochondria and chloroplast membranes was assessed using antibodies directed against AHA2, SMT1, Atmemb11, V-H+ ATPaseAOX and Tic40, respectively. (PPTX) [file pone.0041985.s005.pptx]

## Slide 1
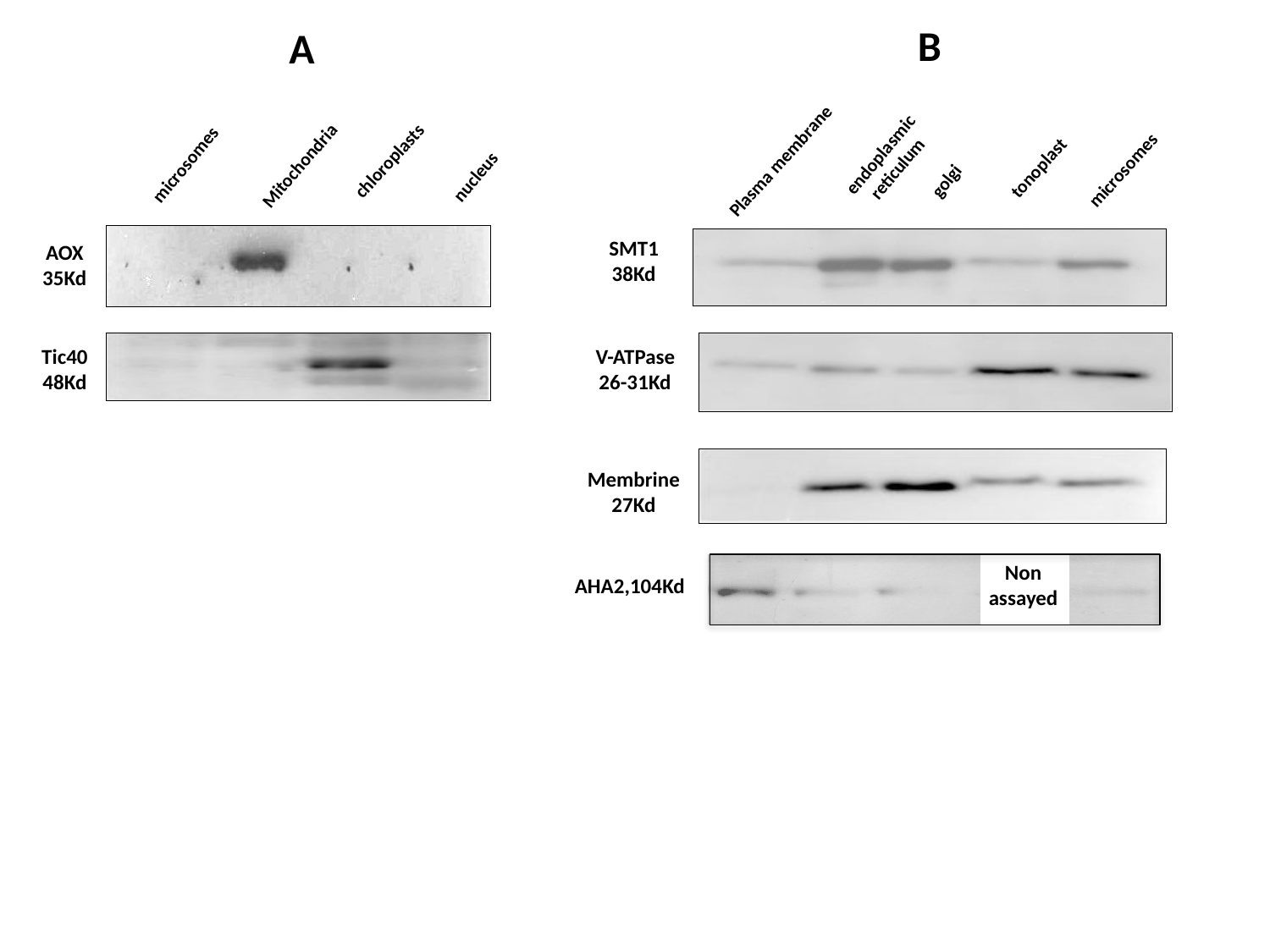

B
A
 endoplasmic
 reticulum
microsomes
Mitochondria
chloroplasts
Plasma membrane
tonoplast
microsomes
nucleus
golgi
SMT1
38Kd
AOX
35Kd
Tic40
48Kd
V-ATPase
26-31Kd
Membrine
27Kd
Non assayed
AHA2,104Kd
