# Supplementary material for: Acyl Chains of Phospholipase D Transphosphatidylation Products in Arabidopsis Cells: A Study Using Multiple Reaction Monitoring Mass Spectrometry
Source: PLoS One. 2012 Jul 25;7(7):e41985. doi: 10.1371/journal.pone.0041985 (PMC3405027; doi:10.1371/journal.pone.0041985)
Supplement: Figure S6 — Glycerophospholipid composition in different membrane fractions. Profiles of PC (A), PE (B), PI (C) and PG (D) as analyzed by MRM mass spectrometry in the different membrane fractions. Lipids were analyzed by mass spectrometry in the MRM mode, searching for the transitions listed in Table 1. (E) Discriminant Analysis of the molecular species according to the lipid class and membrane fractions. (F) The score plot represents 86% of the total variability of molecular species profiles. F1/F2 are the 2 principal eigenvalues for this variability. Variables (phospholipid molecular species)/Factors (F1 and F2) correlations are shown in the loading plot. Each dot in (E) represents the score of a separate profile which comprises the measurements for 15 molecular species indicated in Table B of Figure S6G. The square symbols represent the centroid of the 39 classes indicated in Figure S6G. Each class associates a specific phospholipid (PC, PE, PI, PG) with the extract prepared from a sub-cellular membrane fraction. (G) Tables A (number of repetitions n for each phospholipid class and for each membrane fraction, leading to 225 observations) and B (mean values and standard deviation of the 225 observations for each 15 molecular species). (PPT) [file pone.0041985.s006.ppt]

## Slide 1
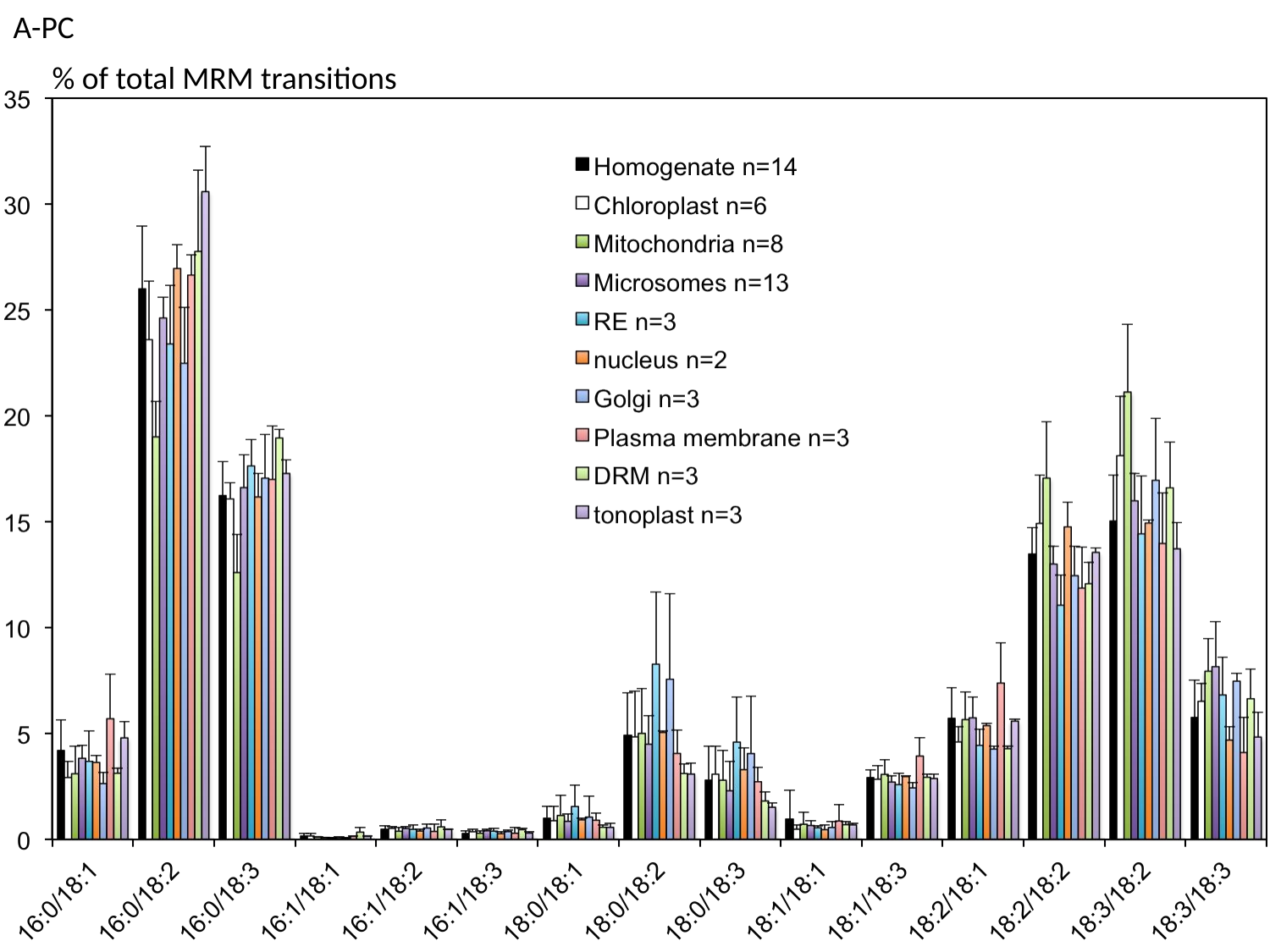

A-PC
% of total MRM transitions

## Slide 2
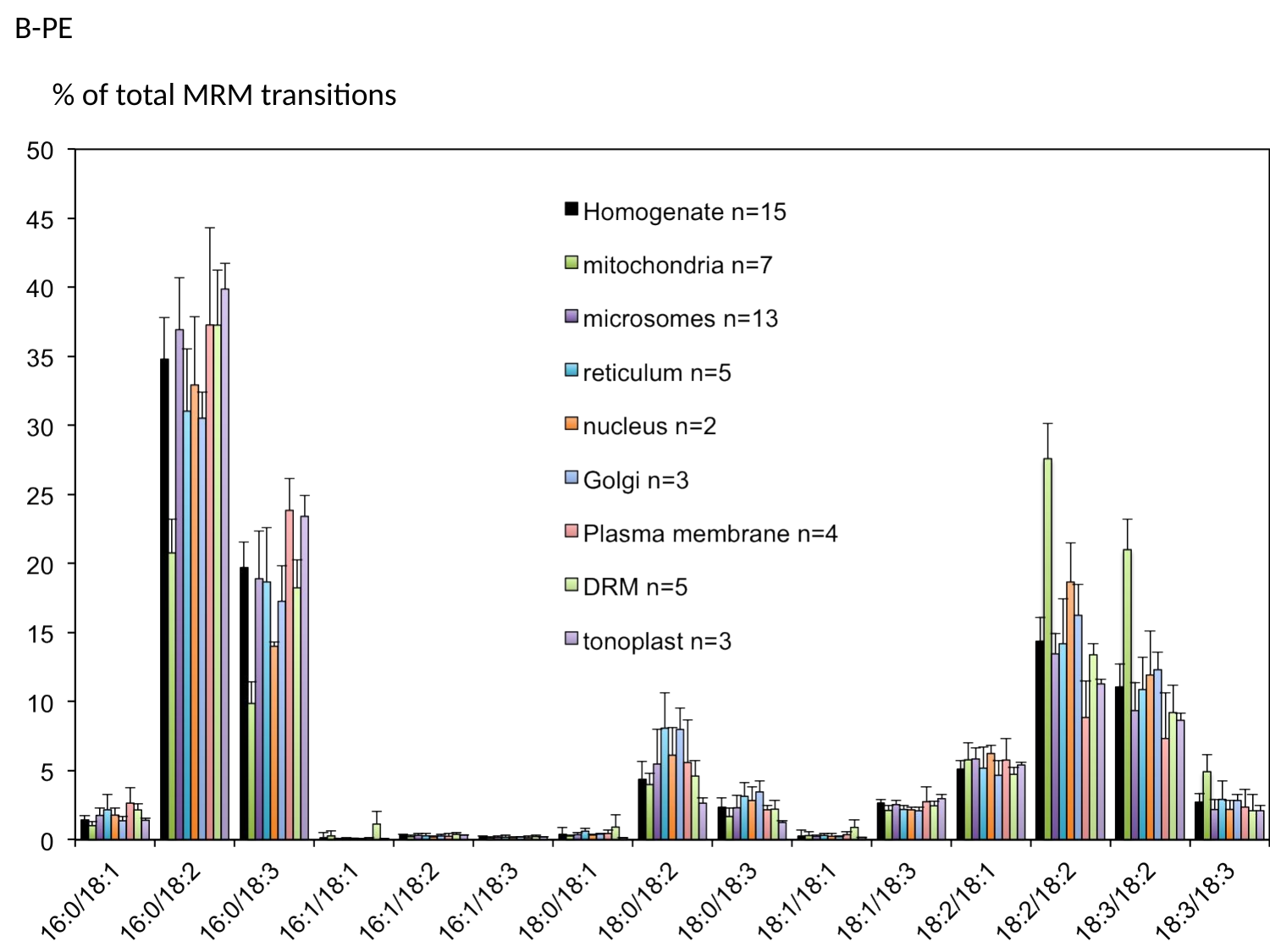

B-PE
% of total MRM transitions

## Slide 3
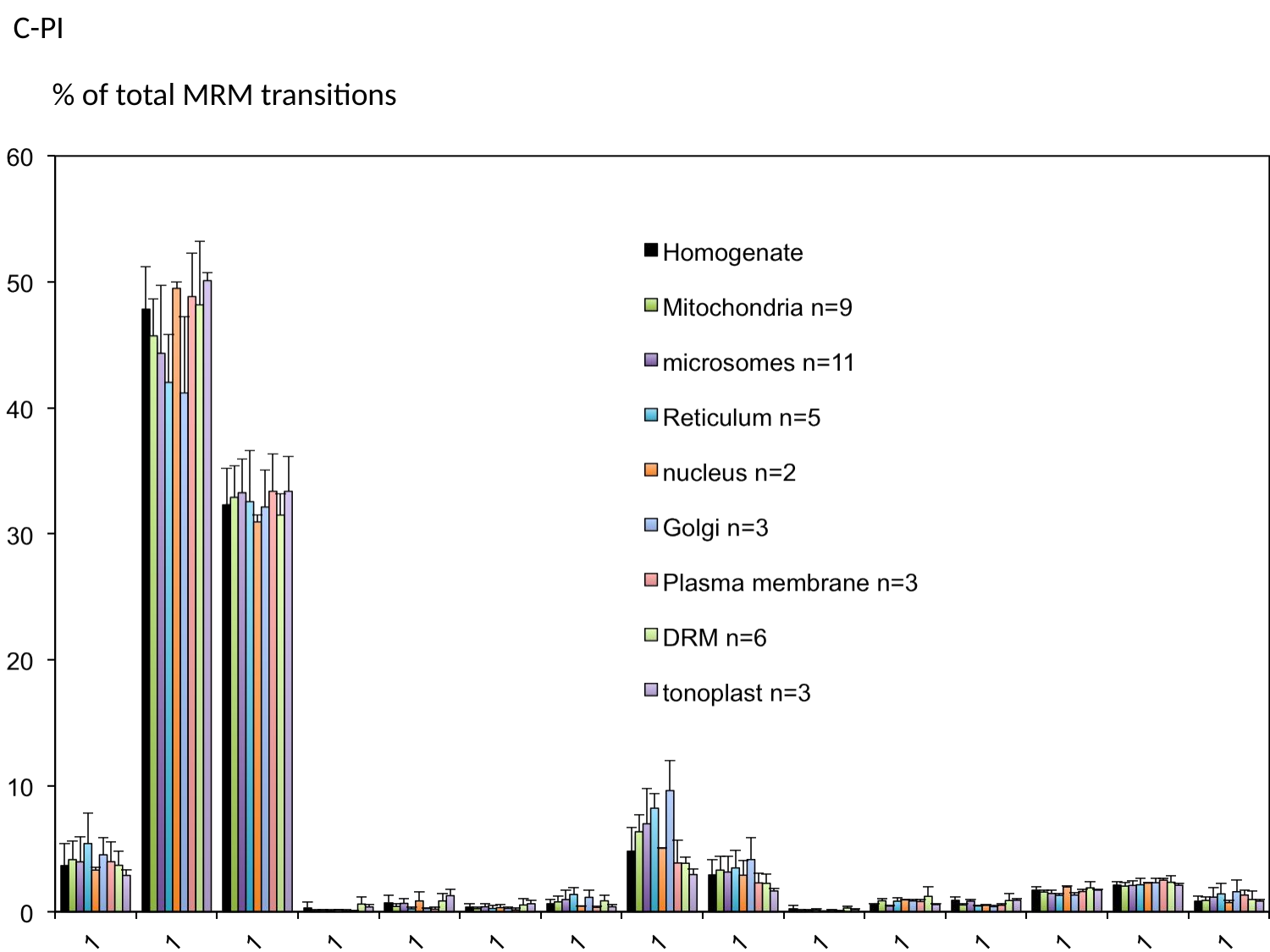

C-PI
% of total MRM transitions

## Slide 4
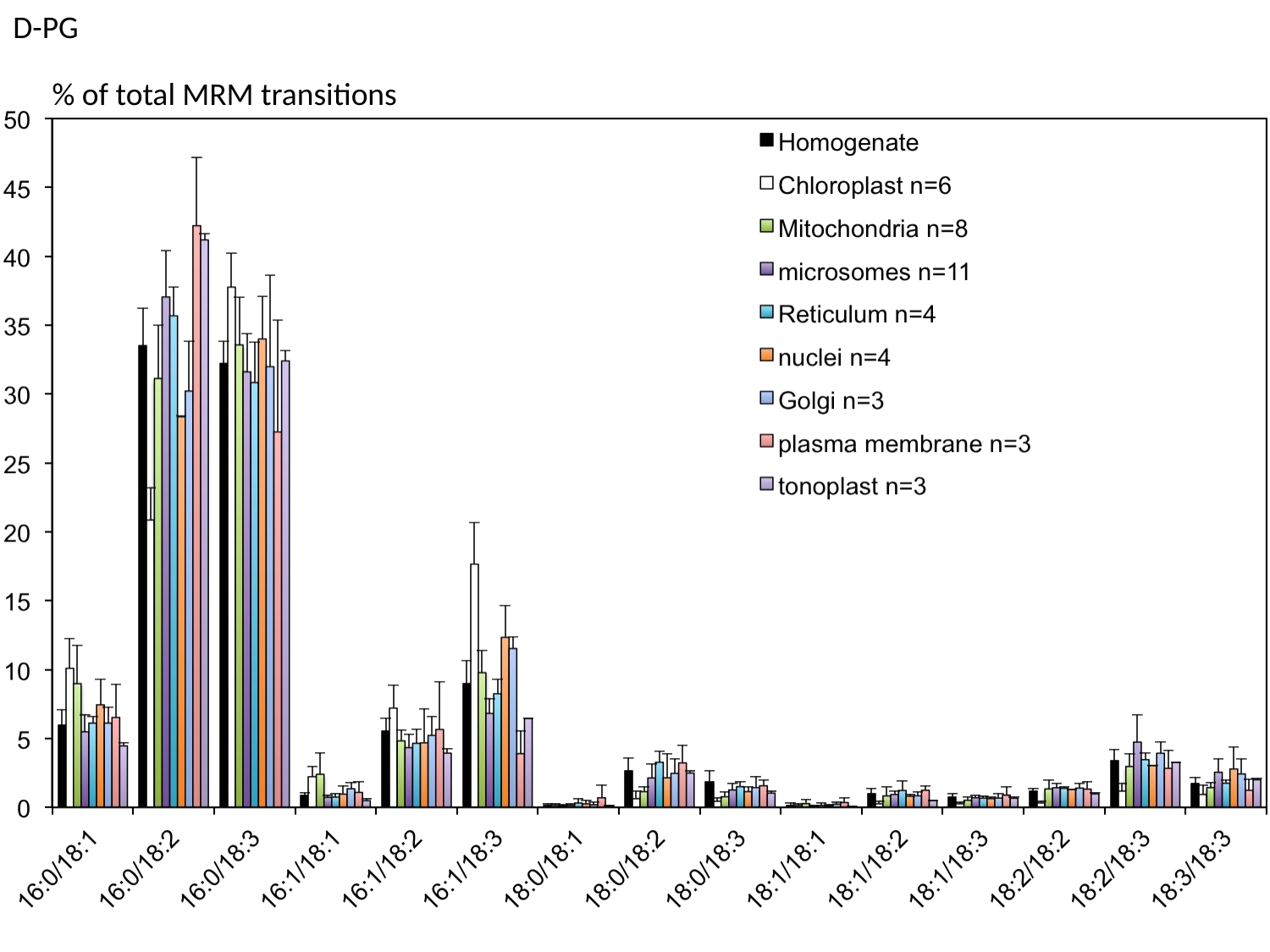

D-PG
% of total MRM transitions

## Slide 5
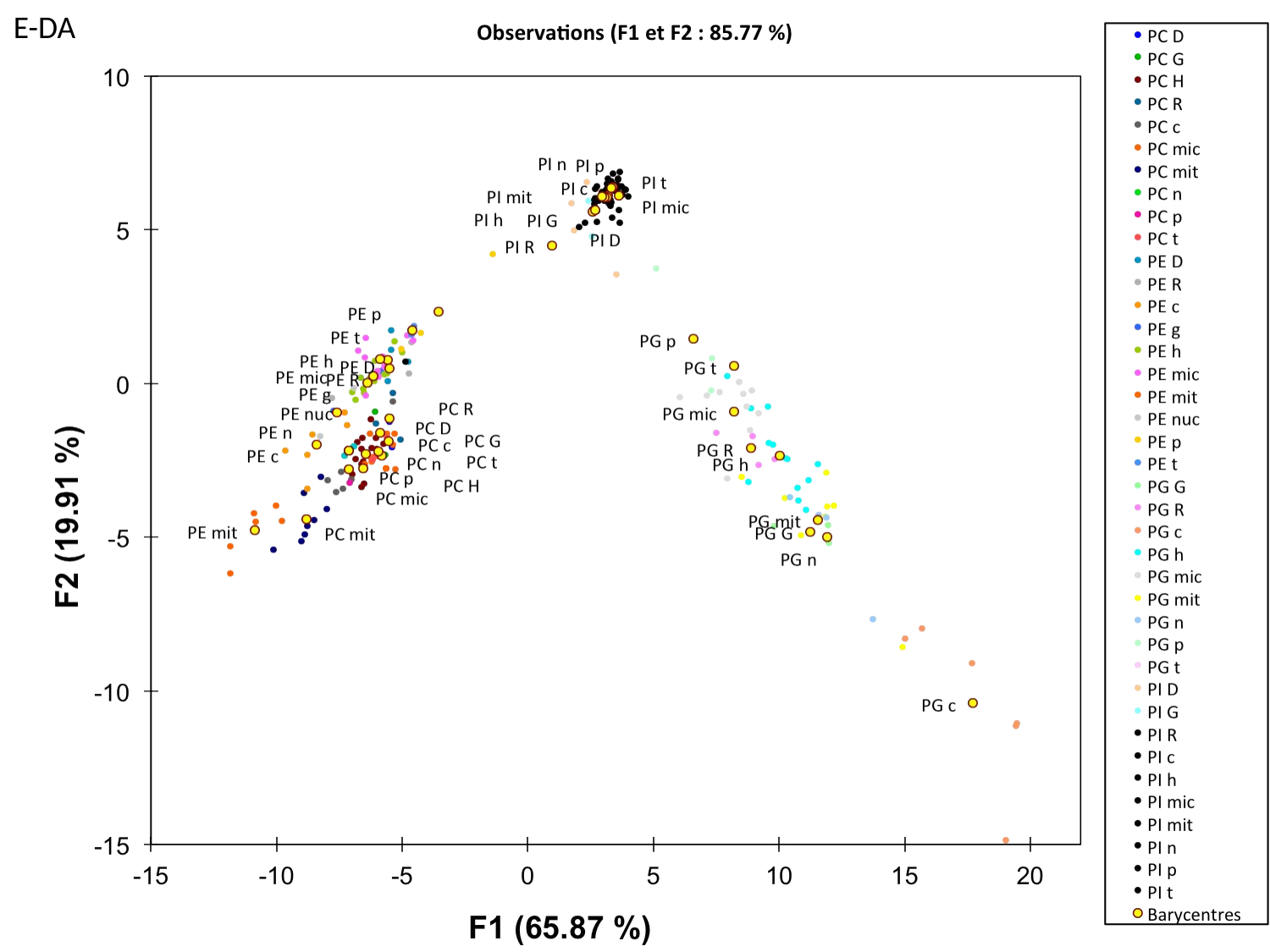

E-DA

## Slide 6
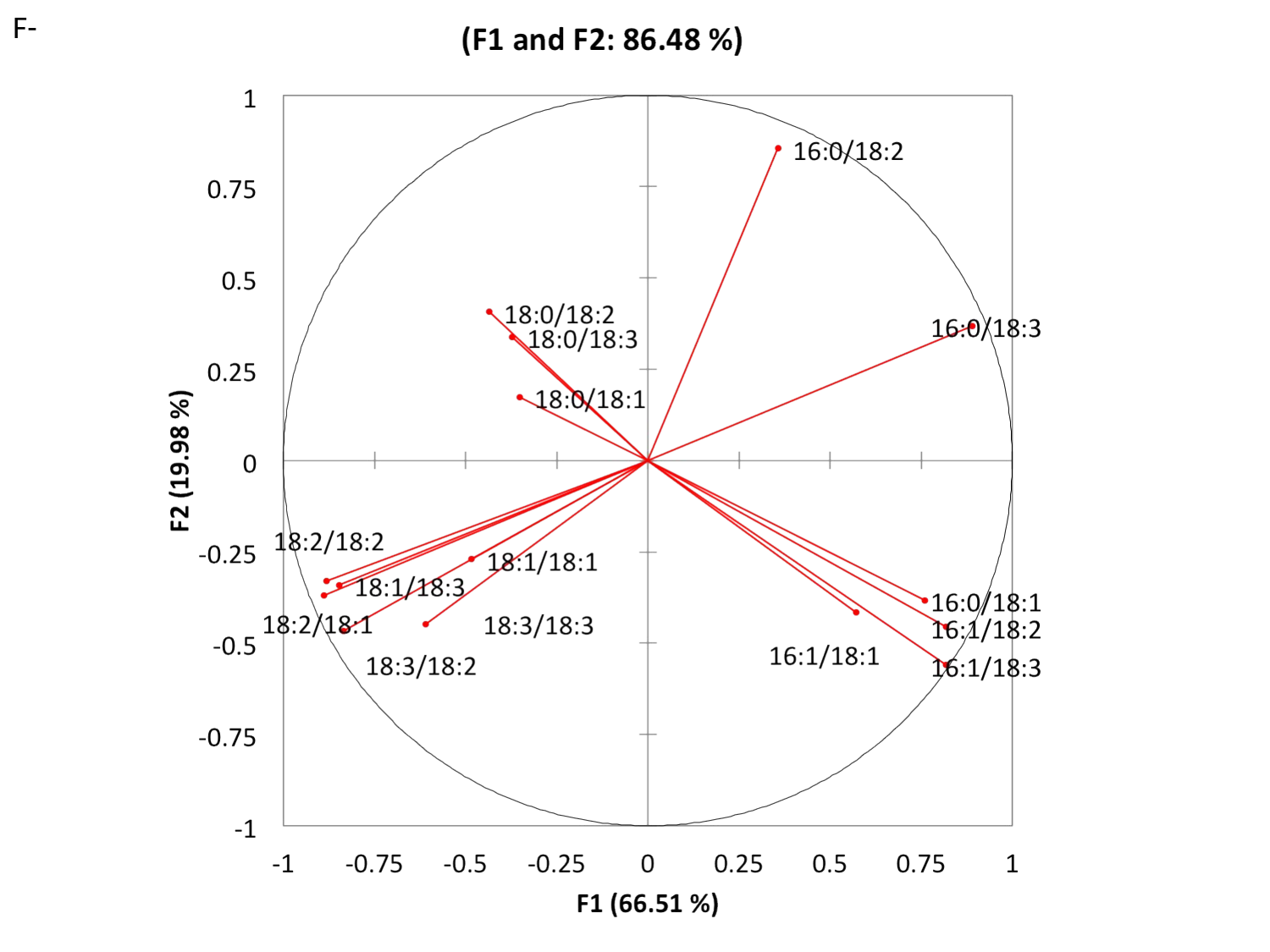

F-

## Slide 7
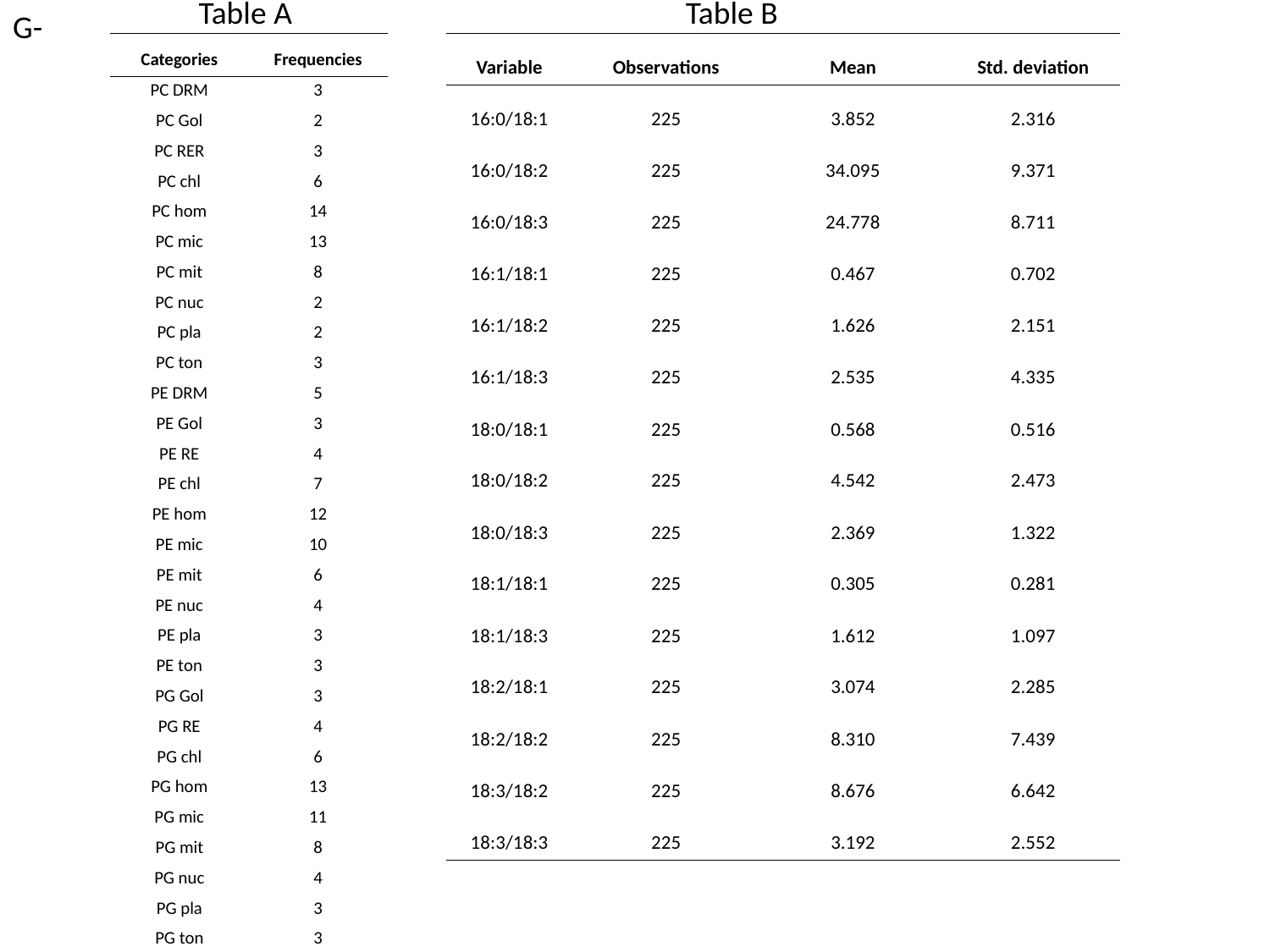

G-
Table A
Table B
| Categories | Frequencies |
| --- | --- |
| PC DRM | 3 |
| PC Gol | 2 |
| PC RER | 3 |
| PC chl | 6 |
| PC hom | 14 |
| PC mic | 13 |
| PC mit | 8 |
| PC nuc | 2 |
| PC pla | 2 |
| PC ton | 3 |
| PE DRM | 5 |
| PE Gol | 3 |
| PE RE | 4 |
| PE chl | 7 |
| PE hom | 12 |
| PE mic | 10 |
| PE mit | 6 |
| PE nuc | 4 |
| PE pla | 3 |
| PE ton | 3 |
| PG Gol | 3 |
| PG RE | 4 |
| PG chl | 6 |
| PG hom | 13 |
| PG mic | 11 |
| PG mit | 8 |
| PG nuc | 4 |
| PG pla | 3 |
| PG ton | 3 |
| PI DRM | 6 |
| PI Gol | 3 |
| PI RER | 4 |
| PI chl | 6 |
| PI hom | 13 |
| PI mic | 10 |
| PI mit | 8 |
| PI nuc | 2 |
| PI pla | 2 |
| PI ton | 3 |
| total | 225 |
| Variable | Observations | Mean | Std. deviation |
| --- | --- | --- | --- |
| 16:0/18:1 | 225 | 3.852 | 2.316 |
| 16:0/18:2 | 225 | 34.095 | 9.371 |
| 16:0/18:3 | 225 | 24.778 | 8.711 |
| 16:1/18:1 | 225 | 0.467 | 0.702 |
| 16:1/18:2 | 225 | 1.626 | 2.151 |
| 16:1/18:3 | 225 | 2.535 | 4.335 |
| 18:0/18:1 | 225 | 0.568 | 0.516 |
| 18:0/18:2 | 225 | 4.542 | 2.473 |
| 18:0/18:3 | 225 | 2.369 | 1.322 |
| 18:1/18:1 | 225 | 0.305 | 0.281 |
| 18:1/18:3 | 225 | 1.612 | 1.097 |
| 18:2/18:1 | 225 | 3.074 | 2.285 |
| 18:2/18:2 | 225 | 8.310 | 7.439 |
| 18:3/18:2 | 225 | 8.676 | 6.642 |
| 18:3/18:3 | 225 | 3.192 | 2.552 |
