# Supplementary material for: Acyl Chains of Phospholipase D Transphosphatidylation Products in Arabidopsis Cells: A Study Using Multiple Reaction Monitoring Mass Spectrometry
Source: PLoS One. 2012 Jul 25;7(7):e41985. doi: 10.1371/journal.pone.0041985 (PMC3405027; doi:10.1371/journal.pone.0041985)
Supplement: Figure S7 — Profiles of PE, PC, PI and PG as analysed by MRM mass spectrometry in Arabidopsis leaves. Lipids were analyzed by mass spectrometry in the MRM mode by searching for the transitions listed in Table 1. The profiles of leaf lipids are shown side by side with that of suspension cell lipids. (PPTX) [file pone.0041985.s007.pptx]

## Slide 1
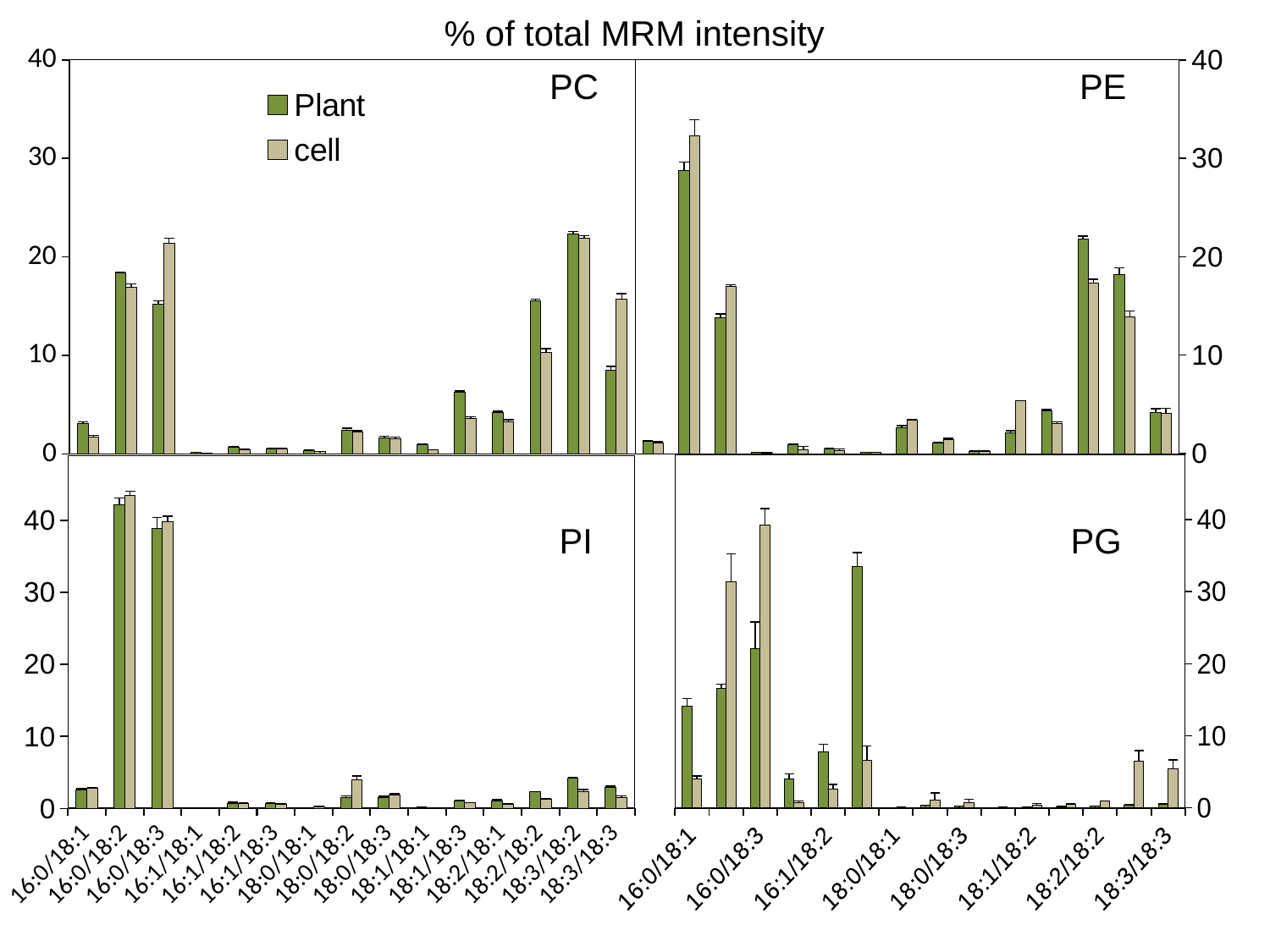

### Chart
| Category | Plant | cell |
|---|---|---|
| 16:0/18:1 | 3.116178400819236 | 1.705192420175907 |
| 16:0/18:2 | 18.38972133264585 | 16.87811410378569 |
| 16:0/18:3 | 15.19964938358446 | 21.36902052055191 |
| 16:1/18:1 | 0.126823537004507 | 0.0630748778036579 |
| 16:1/18:2 | 0.631782124927017 | 0.403211355035375 |
| 16:1/18:3 | 0.513050358793648 | 0.533558993041587 |
| 18:0/18:1 | 0.33806237150761 | 0.20719203543305 |
| 18:0/18:2 | 2.405864488401666 | 2.189965834320716 |
| 18:0/18:3 | 1.575716682174636 | 1.547695197569625 |
| 18:1/18:1 | 0.920408973950885 | 0.375618777663492 |
| 18:1/18:2 | 6.256022444000045 | 3.611008436516591 |
| 18:1/18:3 | 4.213331752265137 | 3.278610206157203 |
| 18:2/18:2 | 15.51007765782553 | 10.30323391744508 |
| 18:2/18:3 | 22.33811512435093 | 21.86821117199255 |
| 18:3/18:3 | 8.46519536774884 | 15.66629215250756 |% of total MRM intensity
### Chart
| Category | PE plant | PE cell |
|---|---|---|
| 16:0/18:1 | 1.223904012888162 | 1.097886949564754 |
| 16:0/18:2 | 28.80123680568026 | 32.31546776313226 |
| 16:0/18:3 | 13.77415254356638 | 17.02065509322049 |
| 16:1/18:1 | 0.134600169605485 | 0.0545625124769935 |
| 16:1/18:2 | 0.92305912631027 | 0.415893053819758 |
| 16:1/18:3 | 0.464359696067111 | 0.32018853137156 |
| 18:0/18:1 | 0.109327886285529 | 0.122844862848641 |
| 18:0/18:2 | 2.595856136001542 | 3.361915099628238 |
| 18:0/18:3 | 1.059654223614294 | 1.393481142935166 |
| 18:1/18:1 | 0.256375180010872 | 0.20091555237768 |
| 18:1/18:3 | 2.148690223806583 | 5.360038463382141 |
| 18:2/18:1 | 4.347641939692843 | 3.017804604468077 |
| 18:2/18:2 | 21.77689927648535 | 17.3364451981868 |
| 18:3/18:2 | 18.20651974351007 | 13.92136493721893 |
| 18:3/18:3 | 4.177723036475264 | 4.060536235368513 |PC
PE
### Chart
| Category | PI plant | PI cell |
|---|---|---|
| 16:0/18:1 | 2.62003795671018 | 2.751864598046399 |
| 16:0/18:2 | 42.1721563587283 | 43.41305911772436 |
| 16:0/18:3 | 38.90767552210388 | 39.74630907259191 |
| 16:1/18:1 | 0.100575915781518 | 0.0736148509960686 |
| 16:1/18:2 | 0.739455034605627 | 0.66699073852326 |
| 16:1/18:3 | 0.721465022711894 | 0.581740180949773 |
| 18:0/18:1 | 0.0458512374117525 | 0.225695280519743 |
| 18:0/18:2 | 1.521642516302067 | 4.01841310427569 |
| 18:0/18:3 | 1.507480619839827 | 1.884278313470604 |
| 18:1/18:1 | 0.116340264802587 | 0.0788399146197727 |
| 18:1/18:3 | 1.059148387714591 | 0.792336095876275 |
| 18:2/18:1 | 1.062568562855075 | 0.622010016688079 |
| 18:2/18:2 | 2.295243030531215 | 1.223536309350619 |
| 18:3/18:2 | 4.20166128339207 | 2.390098822243439 |
| 18:3/18:3 | 2.928698286509475 | 1.531213584123965 |
### Chart
| Category | PG plant | PG cell |
|---|---|---|
| 16:0/18:1 | 14.08389038642821 | 4.038132641318615 |
| 16:0/18:2 | 16.55295885793323 | 31.3530032666441 |
| 16:0/18:3 | 22.10389144545502 | 39.20589905585413 |
| 16:1/18:1 | 4.020506681385806 | 0.737195885315371 |
| 16:1/18:2 | 7.745710127395435 | 2.546934442146593 |
| 16:1/18:3 | 33.48624488204742 | 6.557007721894299 |
| 18:0/18:1 | 0.0352179657813795 | 0.0497075008990951 |
| 18:0/18:2 | 0.340979793373749 | 1.023257421007077 |
| 18:0/18:3 | 0.213845673847279 | 0.724324609485711 |
| 18:1/18:1 | 0.0222036767728874 | 0.0335280319800073 |
| 18:1/18:2 | 0.11970487588717 | 0.422284430575219 |
| 18:1/18:3 | 0.150694873439377 | 0.530368447281086 |
| 18:2/18:2 | 0.2066151109974 | 0.920204851633277 |
| 18:2/18:3 | 0.406992597192841 | 6.458788373073484 |
| 18:3/18:3 | 0.510543052062782 | 5.399363320891912 |PI
PG
