# Supplementary material for: Acyl Chains of Phospholipase D Transphosphatidylation Products in Arabidopsis Cells: A Study Using Multiple Reaction Monitoring Mass Spectrometry
Source: PLoS One. 2012 Jul 25;7(7):e41985. doi: 10.1371/journal.pone.0041985 (PMC3405027; doi:10.1371/journal.pone.0041985)
Supplement: Figure S8 — Cell fractionation protocol. (PPT) [file pone.0041985.s008.ppt]

## Slide 1
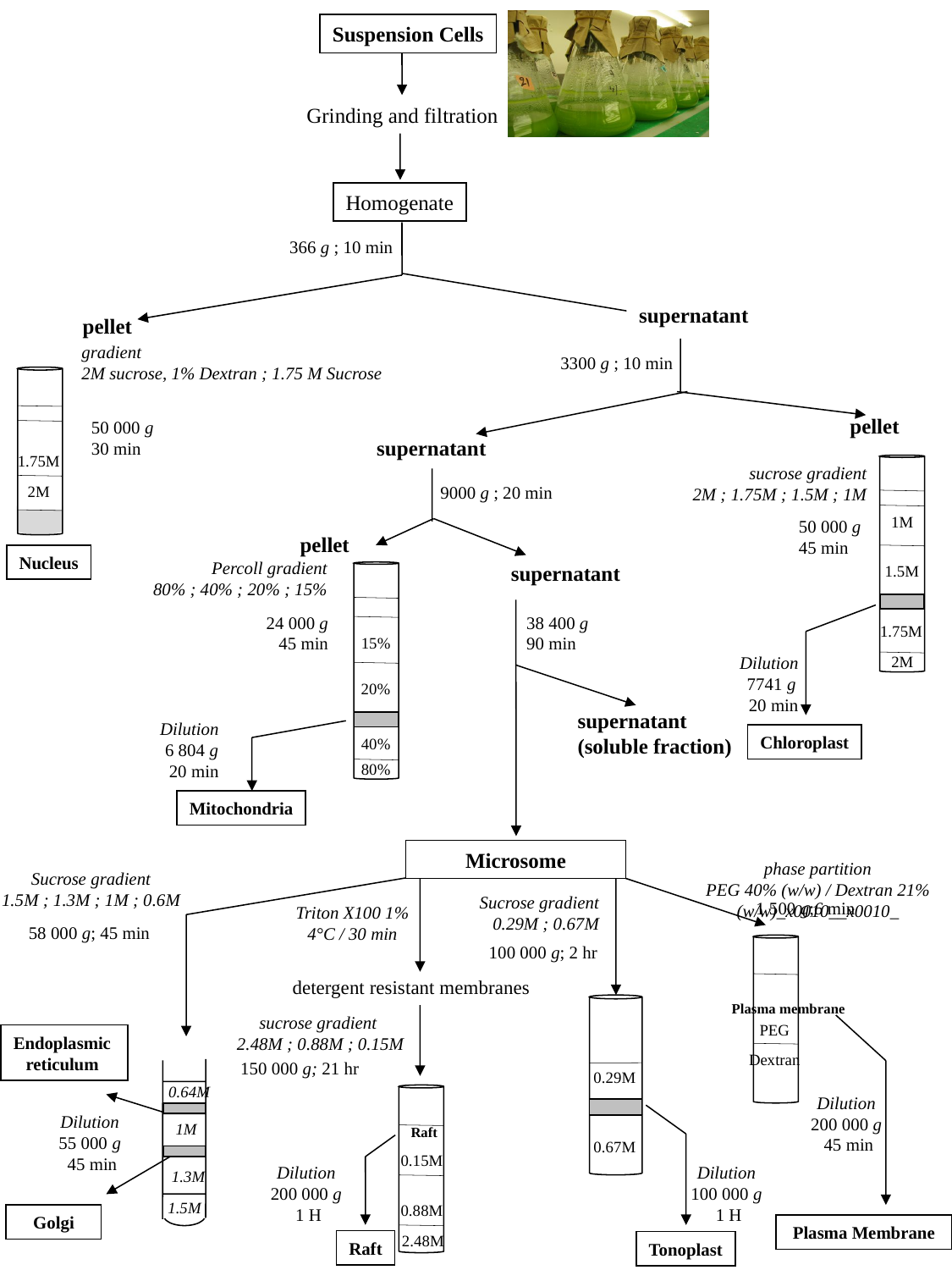

Suspension Cells
Grinding and filtration
Homogenate
 366 g ; 10 min
supernatant
pellet
gradient
2M sucrose, 1% Dextran ; 1.75 M Sucrose
 3300 g ; 10 min
pellet
50 000 g
30 min
supernatant
1.75M
sucrose gradient
 2M ; 1.75M ; 1.5M ; 1M
 9000 g ; 20 min
2M
1M
50 000 g
45 min
pellet
Nucleus
Percoll gradient
 80% ; 40% ; 20% ; 15%
supernatant
1.5M
24 000 g
45 min
 38 400 g
 90 min
1.75M
15%
Dilution
7741 g
 20 min
2M
20%
supernatant
(soluble fraction)
Dilution
6 804 g
 20 min
Chloroplast
40%
80%
Mitochondria
Microsome
phase partition
PEG 40% (w/w) / Dextran 21% (w/w)_x0010__x0010_
Sucrose gradient
1.5M ; 1.3M ; 1M ; 0.6M
Sucrose gradient
 0.29M ; 0.67M
1 500 g;6 min
Triton X100 1%
4°C / 30 min
58 000 g; 45 min
100 000 g; 2 hr
detergent resistant membranes
Plasma membrane
sucrose gradient
 2.48M ; 0.88M ; 0.15M
PEG
Endoplasmic
reticulum
Dextran
150 000 g; 21 hr
0.64M
1M
1.3M
1.5M
0.29M
Dilution
200 000 g
 45 min
Dilution
55 000 g
 45 min
Raft
0.67M
0.15M
Dilution
200 000 g
 1 H
Dilution
100 000 g
 1 H
0.88M
Golgi
Plasma Membrane
2.48M
Raft
Tonoplast
